# UbiN, a novel *Rhodobacter capsulatus* decarboxylative hydroxylase involved in aerobic ubiquinone biosynthesis

**DOI:** 10.1002/2211-5463.13707

**Published:** 2023-09-27

**Authors:** Haruka Nagatani, Yoshiyuki Mae, Miharu Konishi, Motomichi Matsuzaki, Kiyoshi Kita, Fevzi Daldal, Kimitoshi Sakamoto

**Affiliations:** ^1^ United Graduate School of Agricultural Sciences Iwate University Morioka Japan; ^2^ Faculty of Agriculture and Life Science Hirosaki University Japan; ^3^ RIKEN Center for Advanced Intelligence Project Tokyo Japan; ^4^ School of Tropical Medicine and Global Health Nagasaki University Japan; ^5^ Department of Host‐Defense Biochemistry, Institute of Tropical Medicine (NEKKEN) Nagasaki University Japan; ^6^ Department of Biology University of Pennsylvania Philadelphia PA USA

**Keywords:** coenzyme Q, decarboxylative hydrolase, flavin monooxygenase, *Rhodobacter capsulatus*, ubiquinone biosynthesis

## Abstract

Ubiquinone (UQ) is a lipophilic electron carrier that functions in the respiratory and photosynthetic electron transfer chains of proteobacteria and eukaryotes. Bacterial UQ biosynthesis is well studied in the gammaproteobacterium *Escherichia coli*, in which most bacterial UQ‐biosynthetic enzymes have been identified. However, these enzymes are not always conserved among UQ‐containing bacteria. In particular, the alphaproteobacterial UQ biosynthesis pathways contain many uncharacterized steps with unknown features. In this work, we identified in the alphaproteobacterium *Rhodobacter capsulatus* a new decarboxylative hydroxylase and named it UbiN. Remarkably, the UbiN sequence is more similar to a salicylate hydroxylase than the conventional flavin‐containing UQ‐biosynthetic monooxygenases. Under aerobic conditions, *R. capsulatus* Δ*ubiN* mutant cells accumulate 3‐decaprenylphenol, which is a UQ‐biosynthetic intermediate. In addition, 3‐decaprenyl‐4‐hydroxybenzoic acid, which is the substrate of UQ‐biosynthetic decarboxylase UbiD, also accumulates in Δ*ubiN* cells under aerobic conditions. Considering that the *R. capsulatus* Δ*ubiD‐X* double mutant strain (UbiX produces a prenylated FMN required for UbiD) grows as a wild‐type strain under aerobic conditions, these results indicate that UbiN catalyzes the aerobic decarboxylative hydroxylation of 3‐decaprenyl‐4‐hydroxybenzoic acid. This is the first example of the involvement of decarboxylative hydroxylation in ubiquinone biosynthesis. This finding suggests that the C1 hydroxylation reaction is, at least in *R. capsulatus*, the first step among the three hydroxylation steps involved in UQ biosynthesis. Although the C5 hydroxylation reaction is often considered to be the first hydroxylation step in bacterial UQ biosynthesis, it appears that the *R. capsulatus* pathway is more similar to that found in mammalians.

## Graphical abstract

We identified a new flavin monooxygenase, UbiN, in the alphaproteobacterium *Rhodobacter capsulatus*. UbiN catalyzes decarboxylation and hydroxylation reactions in ubiquinone biosynthesis under aerobic conditions. It is the first example of a decarboxylation reaction not catalyzed by UbiD, which is the sole decarboxylase known so far in ubiquinone biosynthesis.

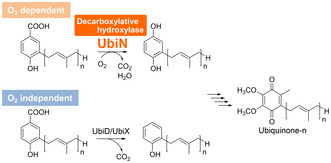

**Abbreviations**
DHB: 3‐decaprenyl‐4‐hydroxybenzoic acid
FMO: flavin monooxygenase
UQ: ubiquinone

Ubiquinone (UQ) is a polyprenylated quinone derivative used as an electron carrier in respiratory and photosynthetic electron transfer chains of alpha, beta, gamma‐proteobacteria and eukaryotes [1]. In bacteria, UQ is mainly synthesized starting with 4‐hydroxybenzoic acid (4‐HB). First, the aromatic ring is polyprenylated and then one decarboxylation, three hydroxylation, and three methylation steps are carried out to yield UQ [2]. This biosynthetic pathway has been established using gammaproteobacterium *Escherichia coli*, and 15 proteins (UbiA to K, T to V, and X) are identified to carry out this synthesis [3]. *E. coli* utilizes three flavin‐containing monooxygenases (FMO), and UbiI, UbiH, and UbiF for the three aerobic hydroxylation reactions of C1, C5, and C6 positions, respectively (Fig. 1A). However, not all three FMO enzymes are conserved in alphaproteobacteria, suggesting that UQ biosynthesis might differ in these species. Using molecular phylogenetic analyses, Pelosi *et al*. [4] have classified UQ‐biosynthetic FMOs into five groups, UbiF, H, I, L, and M, and alphaproteobacteria utilize UbiL, UbiM, or di‐iron protein Coq7 for the three aerobic hydroxylation steps. The hydroxylating positions of *Rhodospirillum rubrum* UbiL and Coq7 as well as that of UbiM from the betaproteobacterium *Neisseria meningitidis* have been demonstrated [4]. However, the correspondence between specific enzymes and their hydroxylation positions remains unknown in some alphaproteobacteria, including *Rhodobacter capsulatus*.

**Fig. 1:**
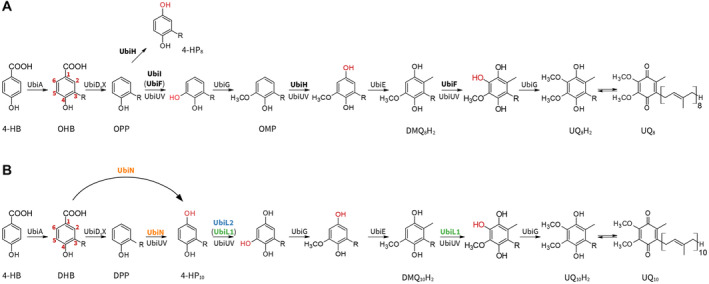
Proposed UQ‐biosynthetic pathways in *E. coli* (A) and *R. capsulatus* (B). Numberings of the ring used in this study were shown in 3‐octaprenyl‐4‐hydroxybenzoic acid (OHB) and 3‐decaprenyl‐4‐hydroxybenzoic acid (DHB). These pathways start with 4‐hydroxybenzoic acid (4‐HB) as ring precursors. Modifications of the rings were carried out in different orders in the pathways. R, polyprenyl tail; OPP, 3‐octaprenylphenol; OMP, 3‐octaprenyl‐5‐methoxyphenol; DPP, 3‐decaprenylphenol; DMQH_2_, demethoxyubiquinol; UQH_2_, ubiquinol; UQ, ubiquinone.

*Rhodobacter capsulatus* is a purple, nonsulfur facultative phototrophic alphaproteobacterium that can grow under aerobic‐dark respiratory or anaerobic‐light phototrophic conditions and uses UQ as its sole quinone source for both growth pathways. A related purple phototrophic alphaproteobacterium *R. rubrum* contains rhodoquinone (RQ) in addition to UQ. RQ is synthesized from UQ by RquA protein and is required under anaerobic growth conditions [5, 6]. Hence, UQ is an essential molecule for even anaerobic growth of *R. capsulatus* and *R. rubrum*, unlike *E. coli* that utilizes menaquinone (MK) under anaerobiosis [7]. Available databases indicate that *R. capsulatus* possesses only two *ubiL* genes for aerobic UQ biosynthesis. Here, we refer to these two *ubiL* products as UbiL1 (WP_013066967.1) and UbiL2 (WP_013069027.1). We found that the co‐expression of both *ubiL1 and ubiL2* (*ubiL1‐L*2) is not able to complement the aerobic growth of *R. rubrum* Δ*ubiL* mutant cells. This lack of complementation of *R. rubrum* Δ*ubiL* mutant cells suggests that *R. capsulatus* might have yet another hydroxylase activity involved in UQ biosynthesis.

Independently of the above‐described experimental findings, a phylogenetically distant FMO (WP_013068139.1) has been detected in the genome of *R. capsulatus* (see below Fig. 2). Remarkably, this FMO is highly similar to salicylate hydroxylase enzymes of the naphthalene degradation pathway, even though the other enzymes of this pathway are not present in *R. capsulatus* genome. This observation suggests that this FMO might not be involved in naphthalene degradation and begs the question of whether it plays a role in UQ biosynthesis. Our results demonstrate that the FMO (WP_013068139.1) is responsible for aerobic C1 hydroxylation during UQ biosynthesis, and we name it UbiN. Moreover, we also analyzed the decarboxylation activity of UbiN because its amino acid sequence shows high similarity to a characterized salicylate hydroxylase, known to catalyze the decarboxylative hydroxylation of salicylate [8, 9]. Our overall results support that UbiN can catalyze the decarboxylative hydroxylation of polyprenylated 4‐hydroxybenzoic acid. Consequently, as the UQ‐biosynthetic decarboxylase UbiD is not always conserved among UQ‐utilizing proteobacteria [3, 10, 11, 12], the decarboxylative hydroxylation carried out by an FMO elucidates the basis of aerobic UQ biosynthesis at least in some species containing UbiN. We conclude that the occurrence of this decarboxylative hydroxylation step implies that *R. capsulatus* UQ biosynthesis is carried out in the same order as mammalian UQ biosynthesis [13].

**Fig. 2:**
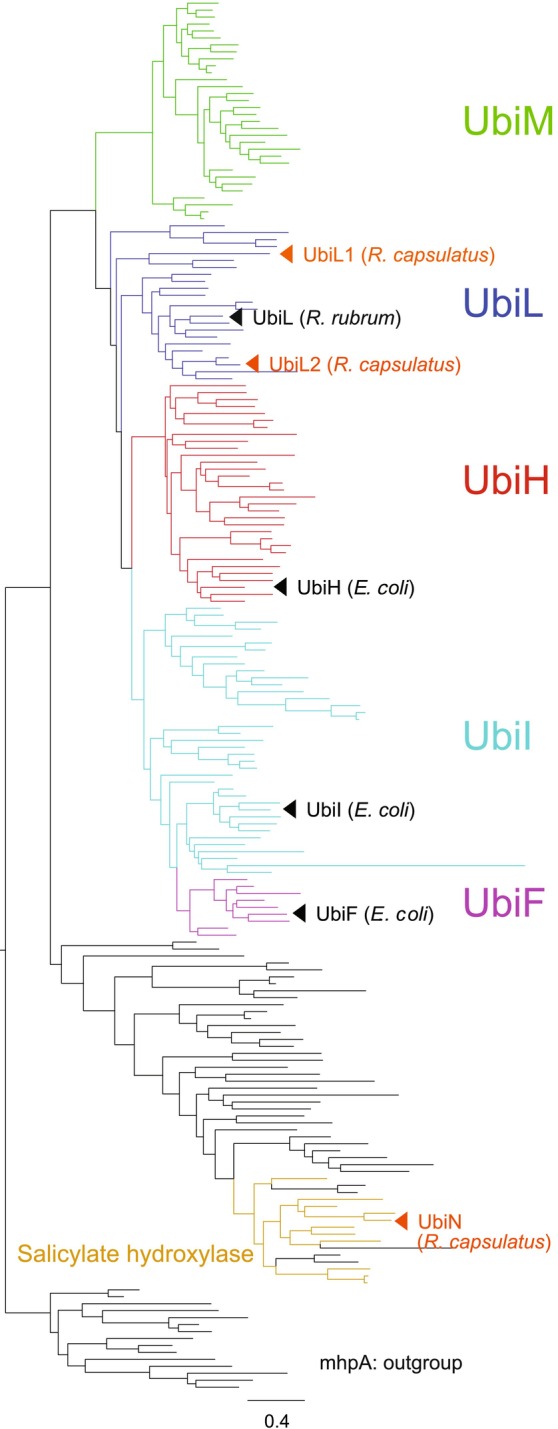
Molecular phylogenetic tree of UQ‐biosynthetic FMOs. Phylogenetic tree of UQ‐biosynthetic FMOs inferred by maximum‐likelihood method under LG + G4 + I + F model. Approximate likelihood‐ratio test values for each branch and sequence identifiers are provided in Fig. S4. The tree was arbitrarily rooted with a cluster containing 3‐(3‐hydroxy‐phenyl)propionate hydroxylases (mhpA; K05712; EC1.14.13.127). Five known classes UbiF, H, I, L, and M were indicated based on Pelosi *et al*. [4]. Sequences assigned to salicylate hydroxylase (K00480; EC1.14.13.9) were highlighted in orange color on their branches.

## Results

### *R. capsulatus ubiL1‐L2* co‐expression is unable to complement aerobic growth of *R. rubrum* Δ*ubiL*

**Fig. 3:**
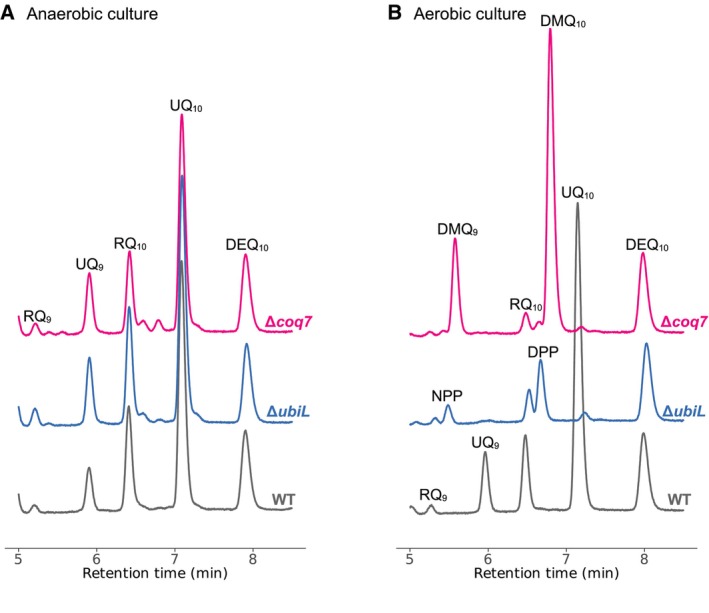
Chromatograms of lipids extracted from *R. rubrum* KO strains. UPLC‐PDA chromatograms of KO strains and WT transferred to aerobic conditions from anaerobic conditions, monitored at a wavelength of 275 nm. (A) *R. rubrum* WT and KO strains have UQ_10_, RQ_10_, and UQ_9_ as major quinones under anaerobic conditions. (B) KO strains accumulated UQ‐biosynthetic intermediates and lost almost all UQ_10_ after transferred to aerobic conditions. Δ*coq7* cells accumulated demethoxyubiquinone‐10 (DMQ_10_) and demethoxyubiquinone‐9 (DMQ_9_). Δ*ubiL* cells accumulated 3‐decaprenylphenol (DPP) and 3‐nonaprenylphenol (NPP). Diethoxyubiquione‐10 (DEQ_10_) was used as an internal standard.

The hydroxylation positions of *R. rubrum* UbiL (C1,5) and Coq7 (C6) are known based on their expression in *E. coli* [4]. We constructed *R. rubrum* Δ*ubiL* and Δ*coq7* strains for complementation assays with *R. capsulatus* genes. These KO strains can synthesize UQ under anaerobic conditions (Fig. 3A), but accumulate UQ‐biosynthetic intermediates under aerobic conditions (Fig. 3B). These results agree with the determined hydroxylation positions of *R. rubrum* UbiL and Coq7. In order to define the hydroxylating positions of two known *R. capsulatus* FMOs UbiL1 and UbiL2, we expressed separately the *ubiL1*, *ubiL2* and also together the *ubiL1‐L2* genes in *R. rubrum* Δ*ubiL* or Δ*coq7* mutant strains lacking the C1, C5‐hydroxylation or C6‐hydroxylation capabilities, respectively (Fig. 3B), and tested their ability to overcome the *R. rubrum* UQ biosynthesis deficiency (Table 1 and Fig. S1). We found that the aerobic growth defect of *R. rubrum* Δ*coq7* was restored by *R. capsulatus ubiL1*, indicating that it was expressed in *R. rubrum* and deduced that UbiL1 catalyzes C6 hydroxylation. Remarkably though, this was not the case for the *R. capsulatus ubiL1* or *ubiL2* expression, or even *ubiL1‐L2* co‐expression in *R. rubrum* Δ*ubiL*. In control experiments, all *R. capsulatus* genes complemented their cognate mutants under appropriate growth conditions (Fig. S2). Thus, *R. capsulatus* might have another UQ‐biosynthetic hydroxylase besides the UbiL1 and UbiL2. Although *R. capsulatus ubiL2* expression in *R. rubrum* was not known at this point unlike *ubiL1* (but see below), these results imply the presence of another UQ‐biosynthetic hydroxylase besides the UbiL1 and UbiL2 in *R. capsulatus*.

**Table 1:** Complementation assays of appropriate *R. rubrum* KO strains by *R. capsulatus ubiL1*, *ubiL2*, and *ubiN* (WP_013068139.1) for aerobic growth. All stains were cultivated at 30 °C for a week under aerobic‐dark conditions. +, grow; −, not grow; EV, empty vector.

Host (*R. rubrum*)	Introduced *R. capsulatus* gene(s)						
	*ubiL1*	*ubiL2*	*ubiN* ^a^	*ubiL1 ubiL2*	*ubiN ubiL1*	*ubiN ubiL2*	EV
Δ*ubiL*	−	−	−	−	+	+	−
Δ*coq7*	+	−	−	+	+	−	−

^a^ ubiN corresponds to WP_013068139.1.

### A remote FMO homolog of *R. capsulatus*

Homology searches were performed to detect the candidate hydroxylase of *R. capsulatus*. Using *R. rubrum* UbiL as queries, the DELTA‐BLAST (Domain Enhanced Lookup Time Accelerated‐BLAST) search identified another FMO in *R. capsulatus* genome while the BLASTP (protein–protein BLAST) failed to detect this FMO [14]. Initially, this FMO has been annotated as salicylate hydroxylase in some databases, due to its sequence similarity to the known salicylate hydroxylase NahG (Table 2 and Fig. S3). Moreover, as a Coq7 homolog was not detected in *R. capsulatus* with any homology search program, we inquired whether this FMO (thereafter named UbiN) could be a candidate UQ‐biosynthetic hydroxylase. A phylogenetic tree of bacterial FMOs shows that UbiN is distant from any other known UQ‐biosynthetic FMOs (Fig. 2 and Fig. S4).

**Table 2:** Percent identity matrix of FMOs created by muscle 3.8.

		1	2	3	4	5
*R. capsulatus* UbiN	1		29.97	20.65	25.88	22.01
*P. putida* NahG	2	29.97		19.37	21.82	22.86
*R. capsulatus* UbiL1	3	20.65	19.37		31.12	32.06
*R. capsulatus* UbiL2	4	25.88	21.82	31.12		46.27
*R. rubrum* UbiL	5	22.01	22.86	32.06	46.27	

### UbiN is involved in UQ biosynthesis

To test the participation of UbiN in UQ biosynthesis, *ubiN*, *ubiN‐L1*, and *ubiN‐L2* from *R. capsulatus* were (co‐)expressed in *R. rubrum* Δ*ubiL* and Δ*coq7* strains, respectively. As a result, both *ubiN‐L1* and *ubiN‐L2* co‐expressions restored the aerobic growth and quinone contents of *R. rubrum* Δ*ubiL* (Table 1, Fig. S1 and Fig. S5). In contrast, the *ubiN* expression alone was unable to restore the aerobic growth of both *R. rubrum* Δ*ubiL* and Δ*coq7* mutants. These results show that UbiN is involved in either C1 or C5 hydroxylation in UQ biosynthesis.

**Fig. 4:**
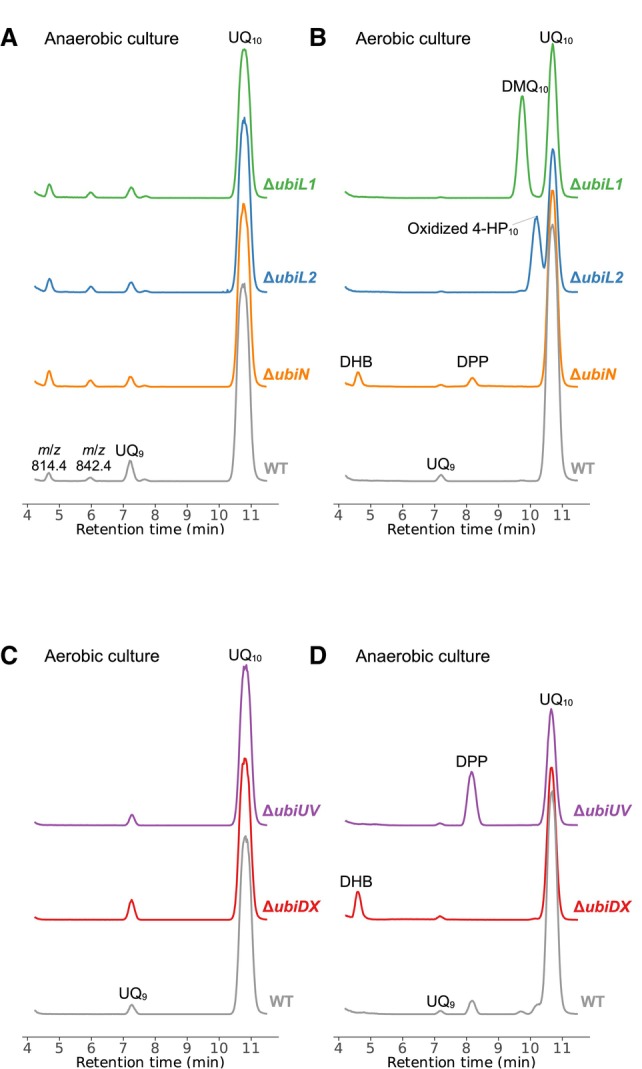
LC–MS ion chromatograms of lipids extracted from *R. capsulatus* KO strains. *R. capsulatus* MT1131 has UQ_10_ as the major quinone and trace amounts of UQ_9_. The extracted ion chromatogram (XIC) range was set for the minimal intermediate 3‐decaprenylphenol (DPP) to UQ_10_ (XIC:790–890). (A, B) Chromatograms of FMO‐gene KO strains and WT transferred to aerobic conditions from anaerobic growth conditions. (A) UQ‐biosynthetic intermediates were not detected. T4.6 (*m*/*z* 814.4) and T6.0 (*m*/*z* 842.4) are not UQ‐related compounds. (B) Δ*ubiL1* cells accumulated demethoxyubiquinone‐10 (DMQ_10_). Δ*ubiL2* cells accumulated 3‐decaprenyl‐4‐hydroxyphenol (4‐HP_10_) detected as its oxidized form. Δ*ubiN* cells accumulated DPP and 3‐decaprenyl‐4‐hydroxybenzoic acid (DHB). (C, D) Chromatograms of Δ*ubiU‐V*, Δ*ubiD‐X* and WT transferred to anaerobic conditions from aerobic conditions. (C) UQ‐biosynthetic intermediates were not detected. (D) Δ*ubiU‐V* and WT cells accumulated DPP, and Δ*ubiD‐X* cells accumulated DHB.

To determine the hydroxylating positions of *R. capsulatus* FMOs, we constructed *R. capsulatus* Δ*ubiL1*, Δ*ubiL2*, and Δ*ubiN* strains, and their aerobic and anaerobic phototrophic growth phenotypes were examined (Table 3 and Fig. S6). The *R. capsulatus* FMO‐gene KO strains ΔubiL1 and ΔubiN were unable to grow under aerobic conditions, while ΔubiL2 grew much slower. The growth defect of Δ*ubiN* cells under aerobic conditions supports the involvement of UbiN in aerobic UQ biosynthesis, while the slow aerobic growth of Δ*ubiL2* cells suggests that another FMO seems to compensate for the UbiL2 activity. As controls, the KO and WT strains were cultured under anaerobic phototrophic conditions and then the cultures were diluted and transferred to aerobic conditions. All quinols in lipid extract were oxidized to quinones with FeCl_3_ prior to injection to eliminate the reduced forms and simplify the chromatograms (Fig. 4A,B). LC–MS analyses of lipids extracted from cells exposed to aerobiosis showed that the Δ*ubiN* cells accumulate 3‐decaprenylphenol (DPP) and 3‐decaprenyl‐4‐hydroxybenzoic acid (DHB) under aerobic conditions (Fig. 4B, orange trace). Considering that polyprenylphenol is a substrate of the first hydroxylase in UQ biosynthesis of *E. coli* and 3‐polyprenyl‐4‐hydroxybenzoic acid a substrate of UQ‐biosynthetic decarboxylase UbiD (Fig. 1A), these accumulation patterns suggest that UbiN catalyzes both the decarboxylation and hydroxylation reactions during aerobic UQ biosynthesis (Fig. 1B).

**Table 3:** Aerobic and anaerobic growth phenotypes of appropriate *R. capsulatus* KO strains. All stains were cultivated for 3 days. ++, grow as WT; +, slow growth; −, not grow.

Strain	Δ*ubiL1*	Δ*ubiL2*	Δ*ubiN*	Δ*ubiD‐X*	Δ*ubiU‐V*	WT
Aerobic	−	+	−	++	++	++
Anaerobic	++	++	++	−	−	++

### Δ*ubiL1* and Δ*ubiL2* cells accumulate DMQ_10_ and 4‐HP_10_ , respectively

Lipid extracts of Δ*ubiL1* and Δ*ubiL2* cells were also analyzed by LC–MS. Figure 4B shows demethoxyubiquinone‐10 (DMQ_10_) accumulation in Δ*ubiL1* cells, indicating the C6 hydroxylation deficiency. This result agrees with the complementation assay that shows that UbiL1 has the C6 hydroxylation activity. Remarkably, Δ*ubiL2* cells accumulated an intermediate (*m*/*z* 811.6) at a retention time of 10.3 min, and the ring structure of the oxidized intermediate was confirmed by LC‐PDA (photodiode arrays detector) analyses (Fig. S7). Additionally, this intermediate was predominantly detected as an unoxidized form (*m*/*z* 813.6) from the lipid extract of Δ*ubiL2* when the FeCl_3_ treatment was omitted (data not shown), indicating that the accumulated intermediate in Δ*ubiL2* cells corresponded to 3‐decaprenyl‐4‐hydroxyphenol (4‐HP_10_). Indeed, a similar accumulation of 4‐HP_8_ in *E. coli* Δ*ubiI* cells has been reported earlier [15].

### UbiN catalyzes decarboxylative hydroxylation

**Fig. 5:**
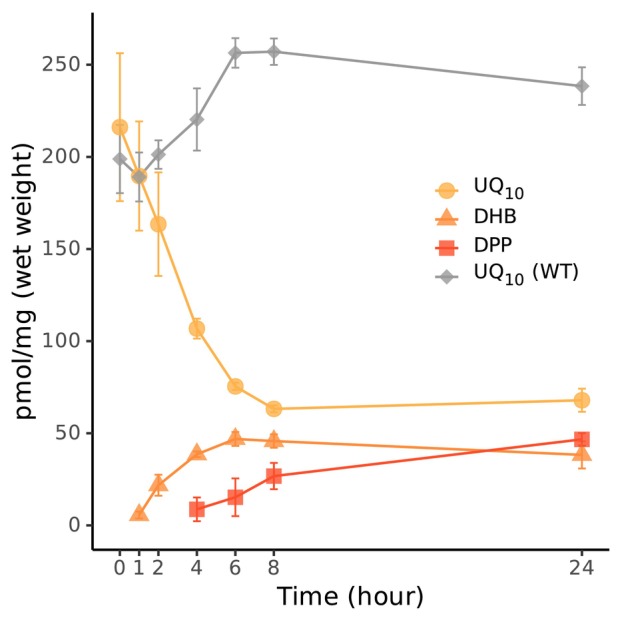
Time course analysis of UQ_10_ and biosynthetic intermediates in Δ*ubiN* cells. UQ_10_, DHB, and DPP were quantified in Δ*ubiN* and WT cells after exposure to aerobiosis at 0, 1, 2, 4, 6, 8, and 24 h. Experiments were repeated four times (*n* = 4), and the error bars are standard deviations.

Homology searches indicated that the UbiN sequence is more similar to salicylate hydroxylases than the UQ‐biosynthetic FMOs (Table 2 and Fig. S3). Salicylate hydroxylase converts salicylic acid into catechol via decarboxylative hydroxylation [8, 9]. Since the Δ*ubiN* cells accumulate the non‐decarboxylated intermediate, UbiN was therefore predicted to catalyze a decarboxylative hydroxylation reaction. To confirm the decarboxylation activity of UbiN, a *R. capsulatus* Δ*ubiD‐X* double mutant strain was constructed as UbiX synthesizes prenylated FMN required as cofactor for UbiD [16]. The *R. capsulatus ubiD* and *ubiX* genes being in the same operon, the *ubiD‐X* double KO strain was obtained using a single kanamycin‐resistance cassette. As another control, the *R. capsulatus* Δ*ubiU‐V* double KO strain was also constructed as the UbiU and UbiV are anaerobic hydroxylases identified in *E. coli* and *Pseudomonas aeruginosa* [17, 18]. Both the *R. capsulatus* Δ*ubiD‐X and* Δ*ubiU‐V* cells show growth defects under anaerobic phototrophic conditions (Table 3 and Fig. S6). The Δ*ubiD‐X and* Δ*ubiU‐V* mutants as well as the WT cells as a control were grown aerobically and then transferred to anaerobic phototrophic conditions, and their lipid extracts were analyzed by LC–MS as before (Fig. 4C,D). Note that the accumulated intermediates have the same retention time and *m/z* as those seen with Δ*ubiN*, and the aromatic structure of the intermediate accumulated in Δ*ubiD‐X* was confirmed by LC‐PDA analyses. Comparison of the UV spectra of the intermediate to that of 4‐hydroxy‐3‐methylbenzoic acid shows that *R. capsulatus* Δ*ubiD‐X* and Δ*ubiN* accumulated the same intermediate, DHB (Fig. S7). In addition, no intermediate accumulation was detected in Δ*ubiD‐X* cells under aerobic conditions (Fig. 4C). Figure 5 shows the time course of UQ_10_ and biosynthetic intermediates contents of Δ*ubiN* and WT cells after exposure to aerobiosis. UQ_10_ decreased and DHB increased in Δ*ubiN* cells, while UQ_10_ level is retained in WT cells. These results demonstrated that UbiN catalyzes the decarboxylative hydroxylation in UQ biosynthesis.

### *ubiN* restored UQ biosynthesis in *E. coli* Δ*ubiD‐H* and Δ*ubiH* strains

**Fig. 6:**
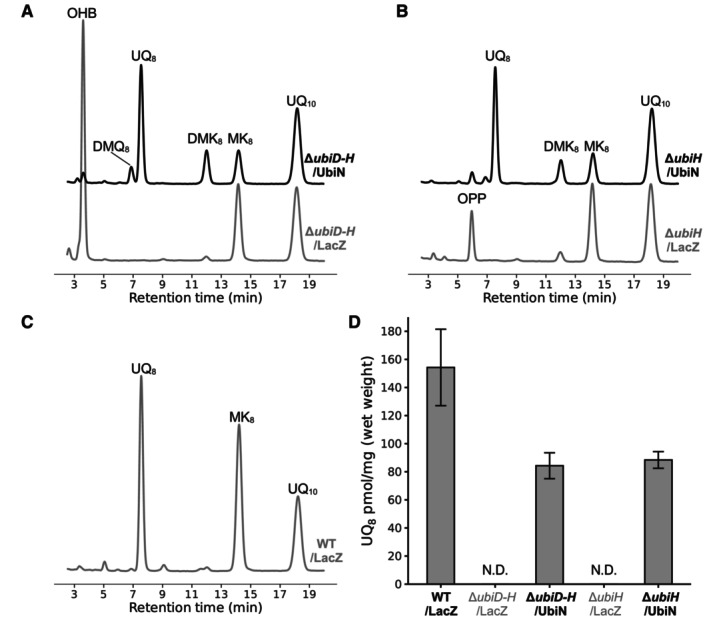
*ubiN* restored UQ_8_ biosynthesis in *E. coli* Δ*ubiD‐H* and Δ*ubiH* cells. (A–C) HPLC‐PDA chromatograms of *E. coli* KO strains and WT transformed with pTrcHis‐ubiN or pTrcHis‐lacZ as a control, monitored at a wavelength of 275 nm. UQ_10_ was used as an internal standard. (A) *ubiN* restored UQ_8_ biosynthesis in Δ*ubiD‐H* double KO strain. Δ*ubiD‐H*/LacZ cells accumulated OHB. (B) *ubiN* restored UQ_8_ biosynthesis in Δ*ubiH* KO strain. Δ*ubiH*/LacZ cells accumulated OPP. (C) WT has UQ_8_ and MK_8_ as major quinones. (D) UQ_8_ of the strains was quantified. Experiments were repeated three times (*n* = 3), and the error bars are standard deviations.

For further confirmation of UbiN activities, the *E. coli ubiD‐H* double KO and *ubiH* KO strains were constructed. *ubiN* was expressed in these KO strains under aerobic conditions and quinone contents were analyzed by HPLC‐PDA. The KO strains transformed with the expression control vector accumulated 3‐octaprenyl‐4‐hydroxybenzoic acid (OHB) and 3‐octaprenylphenol (OPP), respectively, and UQ_8_ was not detected in these KO strains (Fig. 6). As expected, *ubiN* expression restored UQ_8_ biosynthesis in the *ubiD‐H* KO strain. In addition, the *ubiH* KO strain was also rescued by the *ubiN* expression. These results indicate that UbiN can catalyze both decarboxylative hydroxylation of OHB and hydroxylation of OPP.

## Discussion

### Hydroxylating positions of *R. capsulatus* FMOs

The enzyme diversity that occurs among three hydroxylation reactions for aerobic UQ biosynthesis in bacteria has been well described by Pelosi *et al*. [4], demonstrating that one enzyme may catalyze more than one hydroxylation reaction, especially when less than three UQ‐biosynthetic monooxygenases are found in some bacteria, assuming the absence of additional candidates. Starting with this idea, we uncovered a new FMO clade, called UbiN, and experimentally confirmed the occurrence in *R. capsulatus* of three FMOs, UbiL1, UbiL2, and UbiN that are responsible for UQ biosynthesis. As expected, each of the corresponding KO strains accumulated different UQ intermediates, and we concluded that these FMOs have distinct roles in the aerobic UQ biosynthesis, while UbiL1 and UbiL2 belong to the same phylogenetic group.

The accumulation patterns of the UQ intermediates in the mutant cells defined likely specific hydroxylating positions of these three FMOs. *R. capsulatus* Δ*ubiN* cells accumulated both DPP and DHB, like the *E. coli* Δ*ubiH* and Δ*ubiD* cells accumulating 3‐ocataprenylphenol (OPP) and 3‐octaprenyl‐4‐hydroxybenzoic acid (OHB), respectively [19, 20]. As *E. coli* UbiH is considered to catalyze the C1 hydroxylation, *R. capsulatus* UbiN is assumed to catalyze the C1 hydroxylation. However, we note that accumulation of polyprenylphenol might not always represent the C1 hydroxylation deficiency as follows: the 3‐ocataprenylphenol accumulation occurs in *E. coli* Δ*ubiB* and Δ*ubiG* as well as in Δ*ubiH* cells, while UbiB and UbiG are not hydroxylases [21, 22]. To demonstrate firmly that UbiN is C1 hydroxylase, the hydroxylation ability of UbiN was shown by the complementation assays of *R. rubrum* Δ*ubiL* and *E. coli* KO strains. Furthermore, our results show that UbiN catalyzes the decarboxylative hydroxylation of polyprenylhydroxybenzoates. Besides, UbiN also catalyzes the hydroxylation of polyprenylphenols, which is the decarboxylated intermediate (Fig. 1). If UbiN is not able to hydroxylate DPP, then this intermediate would have been accumulated even in the *R. capsulatus* WT cells under aerobic conditions because *ubiD* is expressed [23] and is functional (Fig. 5) under both aerobic and anaerobic conditions [23].

*Rhodobacter capsulatus* Δ*ubiL1* cells accumulated significant amounts of DMQ_10_, demonstrating the C6 hydroxylation activity of UbiL1. When *R. rubrum* Δ*coq7* cells show the growth defect and DMQ accumulations under aerobic conditions (Fig. 3B), the *ubiL1* expression restores the growth defect, further supporting the C6 hydroxylation activity of UbiL1. On the contrary, the *R. capsulatus* Δ*ubiL2* cells accumulated 4‐HP_10_, reminiscent of *E. coli* Δ*ubiI* cells, which have the C5 hydroxylation defect, and accumulate 4‐HP_8_ [15], indicating that UbiL2 is likely to catalyze the C5 hydroxylation. Additionally, the aerobic growth of *R. rubrum* Δ*ubiL* cells was restored by the *ubiN‐L1* co‐expression as well as the *ubiN‐L2* co‐expression, possibly due to the residual C5 hydroxylation activity of UbiL1. Likewise, Δ*ubiL2* cells of *R. capsulatus* show slow growth as compared to WT cells under aerobic conditions, unlike the severe growth defects seen with the Δ*ubiN* and Δ*ubiL1* cells under aerobic conditions. Interestingly, similar to the residual C5 hydroxylase activity seen in *R. capsulatus UbiL*, a residual C5 hydroxylation activity for *E. coli* C6 hydroxylase UbiF has also been reported [15]. However, the molecular phylogenetic analysis based on amino acid sequences indicates that these two C6 hydroxylases are phylogenetically distant FMOs (Fig. 2). Thus, the hydroxylation positions of FMOs are not always correlated with their molecular phylogenetic classifications.

### Remodeling the bacterial UQ‐biosynthetic pathway

The UQ‐biosynthetic pathway contains three hydroxylation steps, and the hydroxylating order was determined in *E. coli*, based on intermediate accumulation analyses. Primarily, the accumulated intermediates in the *E. coli* KO strains are regarded as direct substrates of the gene products. However, 4‐HP_8_ is not regarded as the substrate of UbiI, and the 4‐HP_8_ accumulation is considered to be due to 3‐octaprenylphenol (OPP) hydroxylation activity of UbiH [15]. This interpretation was based on 3‐octaprenyl‐5‐methoxyphenol (OMP) being the original substrate of UbiH. In 1973, Young *et al*. [20] reported that OPP and trace amounts of OMP were accumulated in *E. coli* Δ*ubiH* cells. Until UbiI was identified, UbiB has been annotated as the C5 hydroxylase; thus, OPP was not recognized as the original substrate of UbiH. Based on the idea that UbiN catalyzes the decarboxylative hydroxylation, it is assumed that the carboxylated C1 position is first hydroxylated and then decarboxylated sequentially, because the decarboxylation activity of FMO is initiated by electrophilic aromatic substitution [8, 9]. Hence, the C1 hydroxylation should be the first step of all three hydroxylation steps in *R. capsulatus*. This pathway agrees with the 4‐HP accumulations since the decarboxylative hydroxylation step produces 4‐HP. Although the C5 hydroxylation reaction is often considered to be the first hydroxylation step in bacterial UQ biosynthesis, it appears that the pathway in *R. capsulatus* is more similar to that found in mammalians [13].

### Diversity of decarboxylases in UQ biosynthesis

Our results show that UbiN catalyzes aerobic decarboxylative hydroxylation and infer that UbiD homologs are not essential for aerobic bacteria possessing UbiN homologs. This hypothesis is consistent with the absence of UbiD in some UQ‐producing organisms. Indeed, it is impossible to conclude that all UbiN homologs are involved in the UQ biosynthesis, because substrate specificities of UQ‐biosynthetic FMOs are inconsistent with their amino acid sequence similarity (Fig. 2). Moreover, some bacteria such as *Zymomonas mobilis* lack both *ubiD* and *ubiN* homologs, suggesting the presence of potential decarboxylation activity of C1 hydroxylating FMOs, or unknown decarboxylases. Furthermore, some genera of facultatively anaerobic phototrophs, such as *Rhodomicrobium* and *Rhodovulum* which possess UbiN homologs, lack *ubiD* homologs in their genomes. These phototrophs also lack any homolog of the candidate decarboxylase *ubiZ* [11]. As our results show that the decarboxylation ability of UbiN is limited to aerobic conditions, such facultative anaerobes might possess another anaerobic decarboxylation system. Our next aim will be to investigate the anaerobic UQ biosynthesis in such species.

## Materials and methods

### Bacterial strains and growth conditions

*Rhodobacter capsulatus* strains MT1131 [24] and *R. rubrum* strain S1 = ATCC11170 were grown at 35 and 30 °C, respectively, in Siström's Medium A containing succinate (0.2%) as the carbon source [25]. Fructose was added to a final concentration of 20 mm to *R. rubrum* cultures to promote the growth in aerobic conditions. Anaerobic phototrophic cultivations were performed using GasPak jars (Becton Dickinson, Franklin Lakes, NJ, USA), AnaeroPack (Mitsubishi Gas Chemistry, Tokyo, Japan), and 850 nm LED lamp. Kanamycin (10 μg·mL^−1^), gentamicin (1 μg·mL^−1^), and tetracycline (2.5 μg·mL^−1^) were added when necessary. All *E. coli* strains were grown in Luria–Bertani (LB) medium at 37 °C. To culture UQ‐deficient *E. coli* mutants, glucose or fructose, and potassium phosphate buffer (pH 6.8) were added to final concentrations of 20 and 100 mm, respectively. Kanamycin (50 μg·mL^−1^), gentamicin (12 or 100 μg·mL^−1^ with potassium phosphate buffer), tetracycline (12.5 μg·mL^−1^), carbenicillin (100 μg·mL^−1^), and IPTG (300 μm) were added as needed.

### Gene‐knockout and complementation assays

*Rhodospirillum rubrum* and *R. capsulatus* gene‐knockout (KO) mutants were constructed by homologous recombination, and all constructed KO strains are listed in Table S1. To knockout *coq7* gene in *R. rubrum*, *coq7* gene fragment with 500 bp 5′ upstream and 479 bp 3′ downstream was PCR‐amplified and ligated into pBluescript II KS(+) using XbaI and KpnI restriction sites. A kanamycin‐resistance cassette from pHP45Ω‐Km [26] and double‐digested pBluescript‐coq7 with PspOMI and BlpI were ligated after blunted by T4 DNA polymerase. The *coq7*::*kan* fragment obtained by XbaI and KpnI double digestion was inserted into the SphI site of the suicide vector pZJD29a [27] as blunt end ligation. To knockout *ubi*‐genes, upstream and downstream regions of each gene were separately PCR‐amplified. The fragment sets and a kanamycin‐resistance cassette were combined at the HindIII site and then cloned into the suicide vector pZJD29c at the XbaI and KpnI sites. pZJD29c is a derivative of pZJD29a and constructed by removing the KpnI site of *sacB* gene. These plasmids were introduced into *E. coli* S17‐1 λpir and used as donor strains for conjugation. Liquid cultures of the recipient and the donor stains were mixed on Siström's Medium A agar plate and then incubated for 24 h. The cell mixtures were plated on kanamycin‐containing Siström's Medium A agar plate, and then, kanamycin‐resistant recipients were selected. Replacement of the targeted genomic allele with a kanamycin‐resistance cassette was verified by colony PCR and gentamicin sensitivity, showing the absence of pZJD29c sequence. The monooxygenase‐gene KO strains were purified under anaerobic phototrophic conditions, while the *R. capsulatus ubiU‐V* and *ubiD‐X* KO strains were purified under aerobic conditions to avoid reversions, respectively.

The constructed *R. rubrum* KO strains were transformed with *R. capsulatus* FMO genes and used for the complementation assays. *R. capsulatus* FMO genes and empty vector (pRK415 [28]) as control were introduced via conjugation. pRK2013/*E. coli* HB101 was used as the mobilization helper [28]. After conjugations, tetracycline‐resistant recipients were selected, verified by colony PCR, and purified under anaerobic phototrophic conditions.

*Escherichia coli ubiD* KO mutants were constructed using BW25113, obtained from NBRP (NIG, Japan): *E. coli*, as a parental strain and by the same recombination strategy used for *R. capsulatus*. Deletions of *ubiH* were carried out for BW25113 and the *ubiD* KO strain. The exact ORF of *ubiH* was removed without any antibiotic resistance cassette not to affect the expression of *ubiI*, because *ubiH* and *ubiI* form a gene cluster. First, approximately 500 bp of upstream and downstream regions of the *ubiH* gene were separately PCR‐amplified. Then, these two fragments were combined at the EcoRI site and then cloned into the suicide vector pZJD29c at the XbaI and BamHI sites. After the plasmid was introduced into BW25113 and the *ubiD* KO strain, single‐crossover integrant strains were selected using gentamicin resistance. The double‐crossover stains were obtained by *sacB* counter selection. All transformations of *E. coli* were performed by electroporation, and all *E. coli* mutants were constructed and cultured under anaerobic conditions using AnaeroPack to prevent reversion.

### Cloning and plasmid constructions

*Rhodobacter capsulatus ubiN*, *ubiL1*, and *ubiL2* containing regions were separately PCR‐amplified from genomic DNA. Primer sets were designed to produce fragments that include promoter regions and excluding other ORFs. Desired two‐fragment pairs were combined by T4 DNA kinase and ligase for co‐expression. The single gene fragments or the dual gene fragments were cloned into the pRK415 vector at the XbaI and KpnI sites. These plasmids were introduced into *E. coli* HB101 and sequenced. *R. capsulatus ubiN* was cloned into pTrcHis‐TOPO vector (Invitrogen, CA, USA) for expression in *E. coli*. All primers and plasmids used in this study are listed in Tables S2 and S3, respectively.

### *In silico* protein search and phylogenetic analyses

Using *R. rubrum* UbiL (WP_011391454.1) and *Pseudomonas putida* NahG (WP_011475386.1) sequences as query, blastp and DELTA‐blast searches (blast 2.13.0+) were performed against the predicted proteome of *R. capsulatus* to detect FMOs on NCBI server.

FMO sequences used for the phylogenetic analysis were obtained from the 67 strains used in Pelosi *et al*. [4]. First, all possible orthologues were obtained by blastp version 2.5.0 [29] using all FMO enzymes provided in Table S1 of Pelosi *et al*. [4] as query and an e‐value threshold of 1e‐10. Resultant sequences were screened for having FAD‐binding motifs by hmmsearch version 3.1b2 [30] with PF01494 model and were aligned using muscle version 3.8.31 [31]. After removing partial sequences and trimming ambiguous sites, the matrix composed of 200 sequences and 157 sites was used for phylogenetic analysis. Phylogenetic analysis was performed by phyml‐sms web service [32, 33]. We used LG substitution matrix with supposing 4‐category discrete‐gamma‐distributed rates with invariant sites and using empirical equilibrium frequencies (LG + G4 + I + F). The sequences in the phylogenetic tree were assigned to KEGG Orthology by blastkoala web service [34]. All FMOs used for the phylogenetic analyses are listed in Table S4.

### Qualitative analysis for identification of UQ‐biosynthetic intermediates

Overnight anaerobic cultures of *R. capsulatus* FMO‐gene KO strains and WT were inoculated into 5 mL of Siström's Medium A in 15 mL tubes for OD_630_ = 0.05 and then cultured under anaerobic phototrophic conditions for 16 h. One milliliter aliquots of the cultures were diluted six times in 50 mL tubes with the medium and cultured under aerobic‐dark conditions with shaking at 130 r.p.m. for 4 h. Overnight aerobic cultures of *R. capsulatus* Δ*ubiD‐X*, Δ*ubiU‐V*, and WT were inoculated into 5 mL medium in 50 mL tubes for OD_630_ = 0.05 and then cultured under aerobic‐dark conditions with shaking at 130 r.p.m. for 16 h. One milliliter aliquots of the cultures were diluted six times in 15 mL tubes with the medium and cultured under anaerobic phototrophic conditions for 4 h. The aerobic and anaerobic *R. capsulatus* cultures were centrifuged, and wet weights were determined. Cell pellets were stored at −30 °C until use. Lipid extractions were performed by adding 20 μL 2‐propanol against 1 mg cell pellets, vortexing, and sonication. Lipid extracts were obtained through centrifugation and filtration by a 0.2 μm filter. Oxidation of quinols to quinones in lipid extracts was achieved by adding FeCl_3_ to a final concentration of 2.4 mm. Four microliter aliquots were used for quinone contents analyses by HPLC‐ESI‐TOF‐MS (Nanofrontier LD, Hitachi High‐Technologies, Tokyo, Japan). All HPLC analyses were performed on a C18 reversed‐phase column (Inertsil ODS‐3, 2 μm, 2.1 × 5.0 mm, GL Sciences, Tokyo, Japan). A mixture of 80% methanol, 20% 2‐propanol, and 0.1% formic acid was used as mobile phase at a flow rate of 0.2 mL·min^−1^. UQ and their derivatives were mainly observed as [M + Na]^+^ with our LC–MS system.

UQ‐biosynthetic intermediates were identified by HPLC‐PDA analyses on the same column as HPLC–MS analyses. One hundred microliter aliquots of oxidized lipid extracts were condensed into approx. Twenty microliter by N_2_ gas spray and then 4 μL aliquots were used. A mixture of 90% methanol, 10% 2‐propanol, and 0.1% formic acid was used as a mobile phase at a flow rate of 0.2 mL·min^−1^. *p*‐toluquinone, 4‐hydroxy‐3‐methylbenzoic acid, and *o*‐cresol were used as standards for the UV spectra. All standards were purchased from the Tokyo Chemical Industry (Tokyo, Japan).

### Quantification of UQ and biosynthetic intermediates

The *E. coli* KO strains transformed with pTrcHis‐ubiN and control strains were precultured under anaerobic conditions for 8 h with 2 mL LB medium (+glucose, +potassium phosphate buffer) in 4 mL tubes and then inoculated into 30 mL LB medium (+fructose, +IPTG, and +potassium phosphate buffer) in 200 mL flasks for OD_600_ = 0.005 and grown aerobically for 16 h with shaking at 180 r.p.m. Lipid extractions were performed after adding 20 μL 2‐propanol containing 5 μm UQ_10_ as an internal standard per 1 mg cell pellets (wet weights). Two hundred and fifty microliter aliquots were dried using centrifugal evaporator and then dissolved in 25 μL 2‐propanol containing 2.4 mm FeCl_3_. Concentrated lipid extracts were obtained by centrifugation through a 0.2 μm filter. Four microliter aliquots of concentrated extracts were used for quinone contents analyses. Quantification analyses were performed by HPLC‐PDA (275 nm) using the same column and mobile phase used for the identification of UQ‐biosynthetic intermediates in *R. capsulatus*.

Twenty milliliter anaerobic phototrophic cultures of *R. capsulatus* Δ*ubiN* and WT were diluted 12 times in 1 L flasks with the medium and started aerobic culture under dark conditions with shaking at 180 r.p.m. for 24 h. Twenty milliliter aliquots were sampled at 1, 2, 4, 6, 8 and 24 h. Lipid extractions were performed after adding 20 μL 2‐propanol containing 5 μm diethoxyubiquione‐10 (DEQ_10_) as an internal standard per 1 mg cell pellets (wet weights). DEQ_10_ was synthesized in house, essentially as described by Edlund [35]. Four microliter aliquots of oxidized extracts were analyzed by UPLC‐PDA (275 nm) on a C18 reversed‐phase column (ACQUITY UPLC HSS T3, 1.8 μm, 2.1 × 5.0 mm, Waters). Mobile phase and linear gradient conditions are as follows: (A) methanol:H_2_O (3:7), (B) methanol, and (C) 2‐propanol. All three solvents contained 0.1% formic acid. 0–6 min: (A) 10%, (B) 54–18%, and (C) 36–72%. 6–8 min: isocratic. 8–10 min: isocratic, (A) 10%, (B) 9%, and (C) 81%. Flow rate was 0.4 mL·min^−1^. Amounts of DHB and DPP in lipid extracts were estimated using 275 nm absorption of 4‐hydroxy‐3‐methylbenzoic acid and *o*‐cresol in 2‐propanol (0.1% formic acid), respectively. Quinone contents analyses of *R. rubrum* KO strains were performed following the same methods described above except for the growth time. *R. rubrum* KO strains and WT were grown under anaerobic phototrophic conditions for 3 days and then diluted and cultured under aerobic conditions for 36 h.

## Conflict of interest

The authors declare no conflict of interest.

## Author contributions

HN, YM, MK, and KS planned and performed the experiments. MM performed the molecular phylogenetic analyses. HN and KS wrote the manuscript. KK and FD provided bacterial strains and edited the manuscript.

## Supporting information


**Fig. S1.** Complementation assay of *R. rubrum* KO strains under aerobic conditions.
**Fig. S2.** UQ biosynthesis of *R. capsulatus* KO strains were complemented by the cognate genes under aerobic conditions (A) and anaerobic conditions (B). (PDA: 275 nm).
**Fig. S3.** Alignment of FMOs. FAD‐binding regions are highlighted in orange.
A histidine residue crucial for the activity of NahG and the corresponding residue of UbiN are highlighted in blue.
**Fig. S4.** Molecular phylogenetic tree of UQ‐biosynthetic FMOs with annotations.
**Fig. S5.** Quinone contents analysis of complemented *R. rubrum* KO strains under aerobic conditions. (PDA: 275 nm).
**Fig. S6.** Growth test of *R. capsulatus* KO strains.
**Fig. S7.** UV spectrum of UQ‐biosynthetic intermediates and standards.

**Table S1.** Bacterial strains and relevant genotypes.
**Table S2.** Primers used in this study.
**Table S3.** Plasmids used in this study.
**Table S4.** List of FMOs used for the phylogenetic analysis.

## Data Availability

The data that support the findings of this study are available from the corresponding author [sakamok@hirosaki-u.ac.jp] upon reasonable request.
