# Supplementary material for: UbiN, a novel Rhodobacter capsulatus decarboxylative hydroxylase involved in aerobic ubiquinone biosynthesis
Source: FEBS Open Bio. 2023 Sep 27;13(11):2081–93. doi: 10.1002/2211-5463.13707 (PMC10626278; doi:10.1002/2211-5463.13707)
Supplement: Supplementary file 1 — Fig. S1. Complementation assay of R. rubrum KO strains under aerobic conditions. Fig. S2. UQ biosynthesis of R. capsulatus KO strains were complemented by the cognate genes under aerobic conditions (A) and anaerobic conditions (B). (PDA: 275 nm). Fig. S3. Alignment of FMOs. FAD‐binding regions are highlighted in orange. A histidine residue crucial for the activity of NahG and the corresponding residue of UbiN are highlighted in blue. Fig. S4. Molecular phylogenetic tree of UQ‐biosynthetic FMOs with annotations. Fig. S5. Quinone contents analysis of complemented R. rubrum KO strains under aerobic conditions. (PDA: 275 nm). Fig. S6. Growth test of R. capsulatus KO strains. Fig. S7. UV spectrum of UQ‐biosynthetic intermediates and standards. [file FEB4-13-2081-s002.pdf]

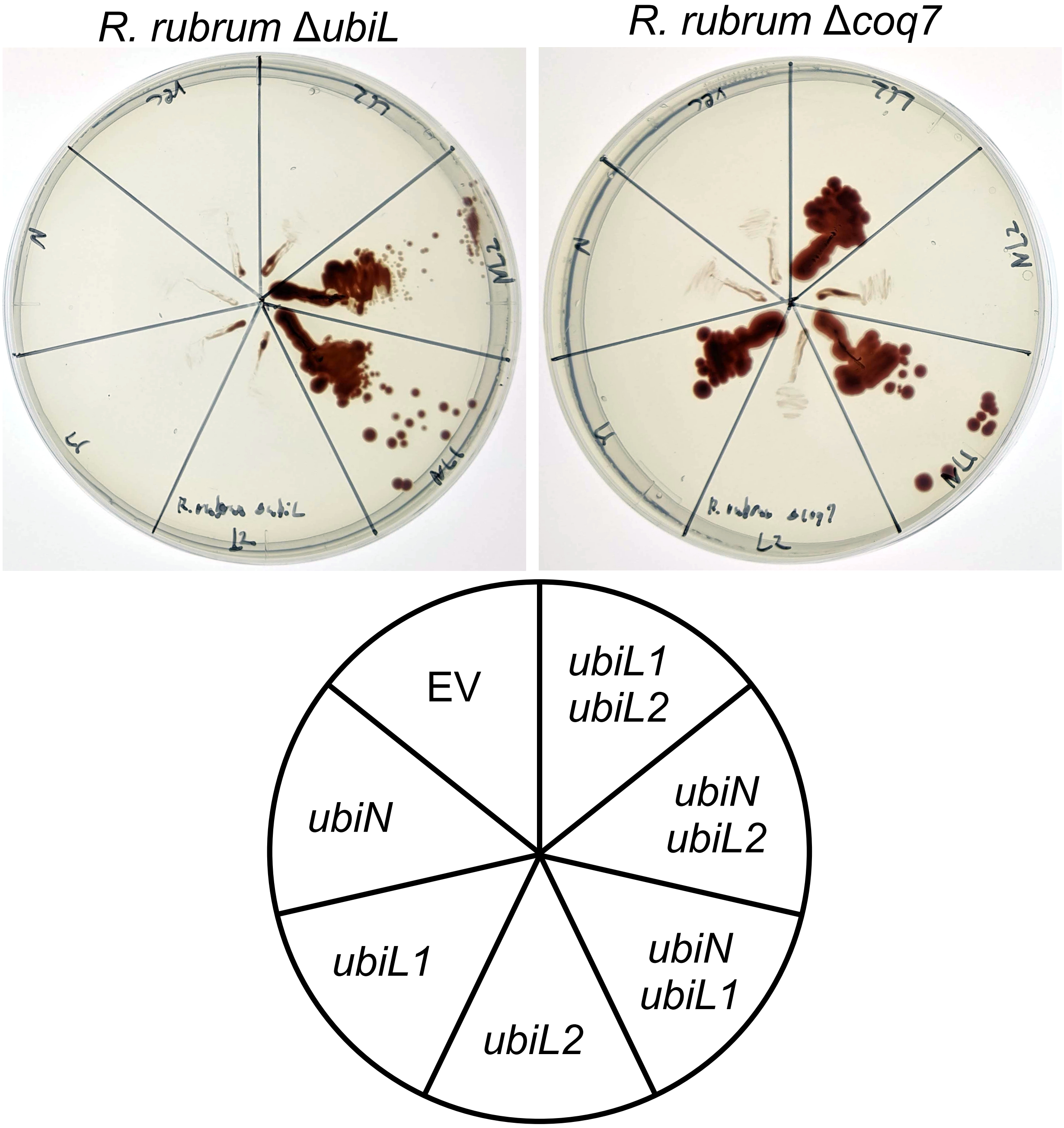

FIGURE S1. Complementation assay of *R. rubrum* KO strains under aerobic conditions.

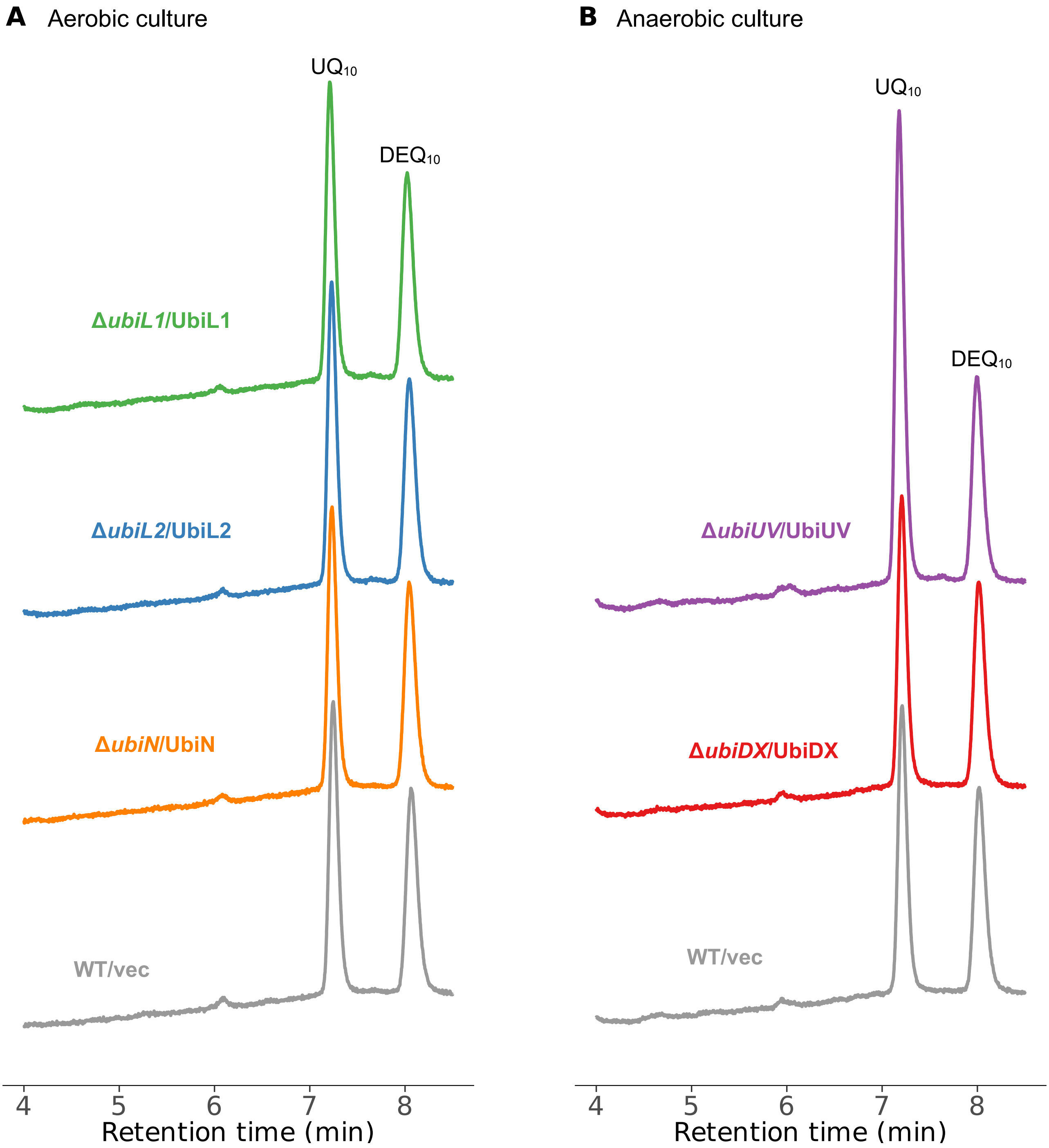

FIGURE S2. UQ biosynthesis of *R. capsulatus* KO strains were complemented by cognate genes under aerobic conditions (A) and anaerobic conditions (B). (PDA:275 nm)

```
WP_013066967.1 UbiL1     ------------------MTETAAPRLSTDILISGGGVAGLVAAAAFGS-AGFTTICVDP
WP_013069027.1 UbiL2     ------------------------MTQVHDVLIAGGGLNGPALALALAG-AGLSVTVIDA
WP_011391454.1 UbiL      MSEPLLRGLAAGDPPSATGPVTGSADKVADVLIVGGGLVGGTLACALAE-KGVSVVVIDG
WP_011475386.1 NahG      ---------------------MKNNKLGLRIGIVGGGISGVALALELCRYSHIQVQLFEA
WP_013068139.1 UbiN      ------------------------MLIGRKVTVLGAGVAGLAVARALAL-RGAEVTVLEQ
                                                       : : *.*: * . *  :       .  .:

WP_013066967.1 UbiL1     EPPVTSEGDAGADMRTTAFLHPSRAVLDRAGL---WARLAPHAAPLQVMRIIDAGGARPE
WP_013069027.1 UbiL2     APAQ-RRADDAFDGRAYALALASQKLLAALGL---WPAVAAQAQPINDVKAFDGRPGEGA
WP_011391454.1 UbiL      EDPE-ALLAAGYDGRCSAIALACQRLLDTIGL---WDLLGGESQPILDIRVVD-----GG
WP_011475386.1 NahG      APAF-GEVGAGVS-----FGPNAVRAIVGLGLGEAYLQVADRTSEPWEDVWFEWRRGSDA
WP_013068139.1 UbiN      ADAI-REVGAGLQ-----ISPNGARVLHALGLGEALAAAGPQAE------AIELREGETG
                           .       . .     :       :   **       .  :        .:

WP_013066967.1 UbiL1     ARIVRDFDAADISDEPFGWNFPNWLLRREMVARLTEIPLVDFRPGVATVGLVTRESGAEV
WP_013069027.1 UbiL2     APFFLHFDSRELDQKPVGYMLEDRFLYRAFLAAMQANPLITLISETSVVAQEVLPGSIRV
WP_011391454.1 UbiL      SPLFLHYAQAE-AQGPMGYMVENRLLRQAILTRLGRLPAATLLAPARMTALRRDLDGVSA
WP_011475386.1 NahG      S-----YLGATIAPG-VGQSSVHRADFIDAL--VTHLPEGIAQFGKRATQVEQQGGEVQV
WP_013068139.1 UbiN      KRVTR-LDLARLRPG-EEYRLLHRARLIELLAEGARAAGVEIKLQSRVAEVMLGPHLPRL
                                                .      :      .          .

WP_013066967.1 UbiL1     RLSDGTLVTAKLVIGADGRNSFVR----EAAGIGTKTTHYGQKALVCAVTHP--------
WP_013069027.1 UbiL2     TLSNGESLSGRVLIGADGRRSGVA----ERAGIGREGWGYGQTALVAALAHE--------
WP_011391454.1 UbiL      TLSDGQTVRARLVVGADGRRSQVR----ESAGIGIRTLGYGQTAIVLTVEHE--------
WP_011475386.1 NahG      LFTDGTEYRCDLLIGADGIKSALRSHVLEGQGLAPQVPRFSGTCAYRGMVDSLHLREAYR
WP_013068139.1 UbiN      KMLKGEEIETGLLIGADGLQSRVR-----RALNGEGRPFFTHQVAWRTLIPC--------
                          : .*      :::**** .* :          .     :        :

WP_013066967.1 UbiL1     ----SPHGNVSTEVHRSGGPFTLVPLPDRDGKPSSAIVW----MERGPEAERLAALAPAD
WP_013069027.1 UbiL2     ----KPHEGIAYQLFMPNGPLAILPLT----GNRSSIVW----SETDANAAVIATLSDDD
WP_011391454.1 UbiL      ----RSHRGCAVEHFLPAGPFAILPMP----GNRSSLVW----TERSDLVPGLLALPAEH
WP_011475386.1 NahG      AHGIDEHLVDVPQMYLGLDGH-ILTFPVRNGGIINVVAFISDRSEPKPTWPADAPWVREA
WP_013068139.1 UbiN      ----DDAEPKVAQVFMGDGRH-LVSYPIGR-GLRNIVA-----VEERQRWTAEG-WSHRD
                                     : .   .   ::. .       . :.      *

WP_013066967.1 UbiL1     FEREMTERSTGV----LGPLTLVTRRALWPM-ITQIACRFSAERVALIAEAAHVIPPIGA
WP_013069027.1 UbiL2     FMELVRPRFGDF----LGRISLVGPRFSYPL-NLTLARAYAADRVALVGDAAHGVHPIAG
WP_011391454.1 UbiL      FQAELERRFGDH----LGWVRPVGPRFSYRL-TLQAANRYVDHRLALVGDAAHGMHPVAG
WP_011475386.1 NahG      SQREMLDAFAGWGDAARALLECIPAPTLWALHDLAELPGYVHGRVVLIGDAAHAMLPHQG
WP_013068139.1 UbiN      DPRSLLAAFEEFCPQVQDWLEAVKEPWLWGLFRHRVARVWQGRGAAILGDAAHPTLPFMA
                             :              :  :     : :        :     .::.:***   *  .

WP_013066967.1 UbiL1     QGLNMSLADLRCLLDL---AEKDPAHLGSAAMLATYNRRRWPEVKAREVGIDFLNRASMV
WP_013069027.1 UbiL2     QGLNLGLRDVAALAEVLIGAMRRGEDIGSIHTLERYQLWRRFDATTLALGMDTVNKLFSN
WP_011391454.1 UbiL      QGMNYGLRDVAVLAERLVAAQRLGLDPGAPALLAEYEALRRPDNLLMLAITDALVRLFSN
WP_011475386.1 NahG      AGAGQGLEDAYFLARLLGDTQADAG--NLAELLEAYDDLRRPRACRVQQTSWETGELYEL
WP_013068139.1 UbiN      QGAVMALEDAWVLAASLAEAQSDAE--G----LAAYQAARRGRCEAIVEAANENARNYHL
                          *   .* *   *      :       .    *  *:  *.

WP_013066967.1 UbiL1     EPRPLRDLR-----AAALGALYAFKPVRKTL----------MRAGLGMR----------
WP_013069027.1 UbiL2     DNPILRAGR-----DLGMGLVQAIGPLRRGF----------MRQAAGL--AGPQ-PKLLQ
WP_011391454.1 UbiL      DIAPVALAR-----RLGIGAVERMGPLKRLF----------MRHAMGTLKLGPEPPRLMR
WP_011475386.1 NahG      RDP--VVGA-----NEQLLGENLATRFDWLWNHDLDTDLAEARARLGWEHGGGGALRQG-
WP_013068139.1 UbiN      SGPARVIGHLALRTASAIAPGKILGRFDWVY---------------GHDVTAPRK-----
                                          :        . .                 *

WP_013066967.1 UbiL1     ----
WP_013069027.1 UbiL2     GRQI
WP_011391454.1 UbiL      GVPL
WP_011475386.1 NahG      ----
WP_013068139.1 UbiN      ----
```

FIGURE S3. Alignment of FMOs. FAD binding regions are highlighted with orange. A histidine residue crucial for the activity of NahG and corresponding residue of UbiN are highlighted with blue.

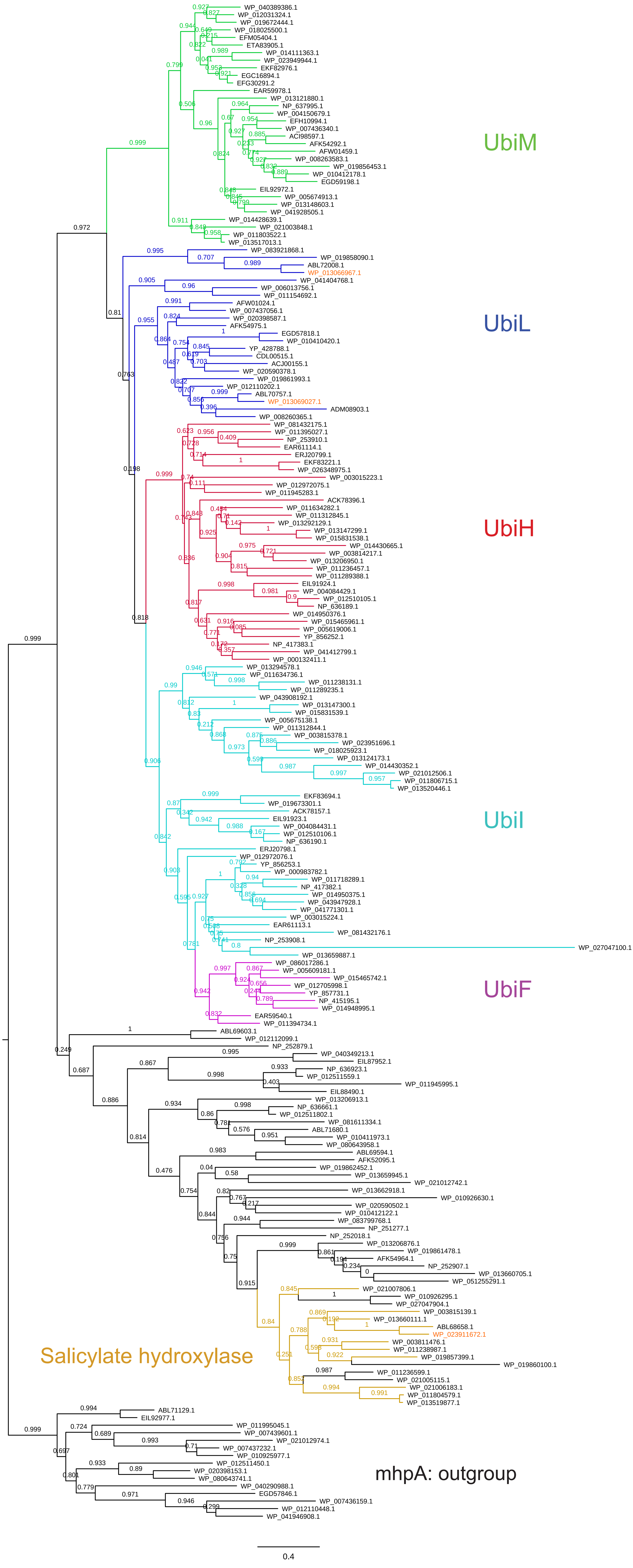

FIGURE S4. Molecular phylogenetic tree of UQ-biosynthetic FMOs with annotations.

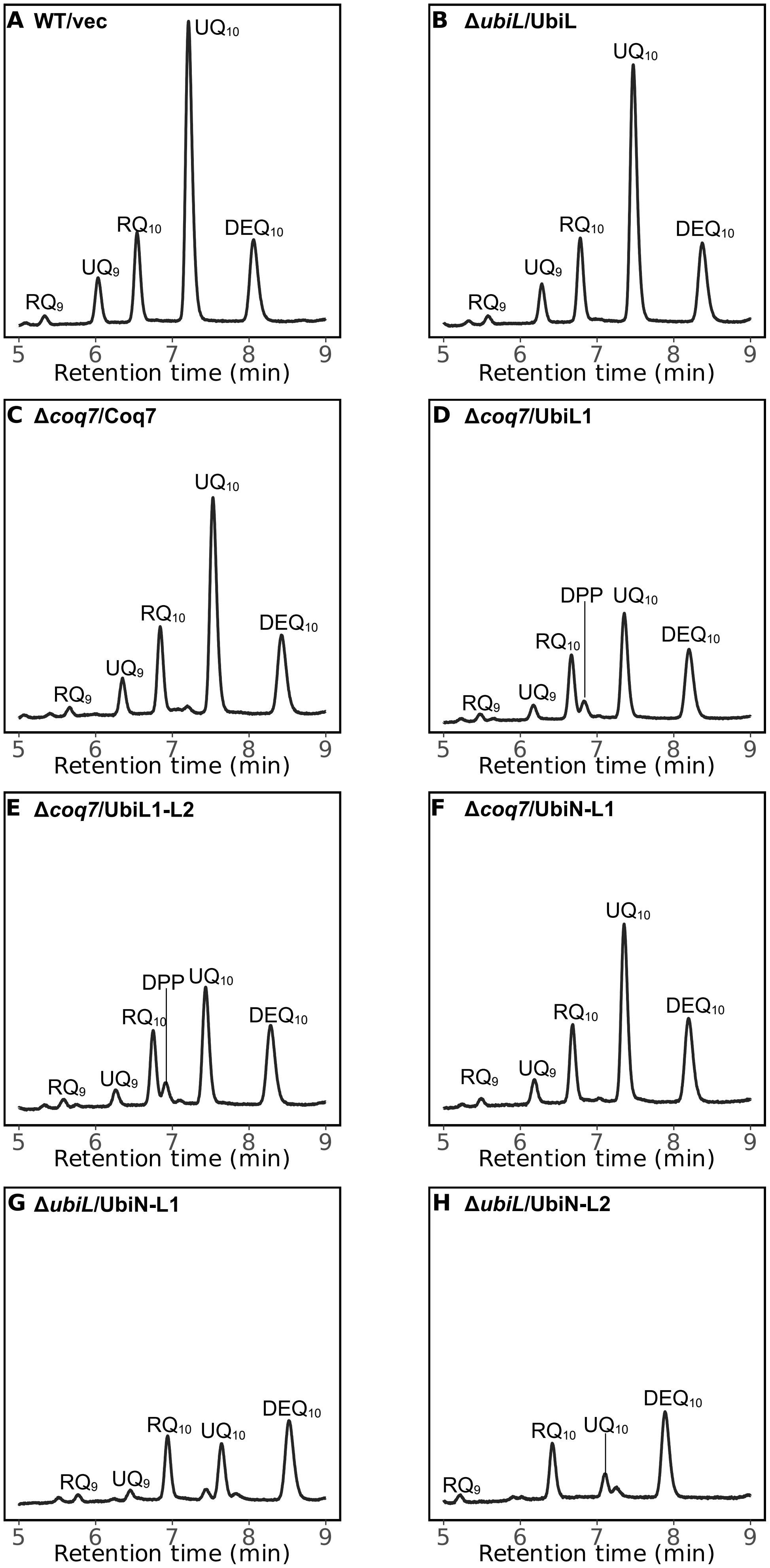

FIGURE S5. Quinone contents analysis of complemented *R. rubrum* KO strains under aerobic conditions. (PDA:275 nm): (A), (B), and (C) are positive control strains. (A) S1 (wild type)/ pRK415. (B) Δ*ubiL*/ pRK415-Rrubrum_ubiL. (C) Δ*coq7*/pRK415-Rrubrum_coq7. (D) Δ*coq7*/pRK415-ubiL1. (E) Δ*coq7*/ pRK415-ubiL1-L2. (F) Δ*coq7*/pRK415-ubiN-L1. (G) Δ*ubiL*/pRK415-ubiN-L1. (H) Δ*ubiL*/pRK415-ubiN-L2.

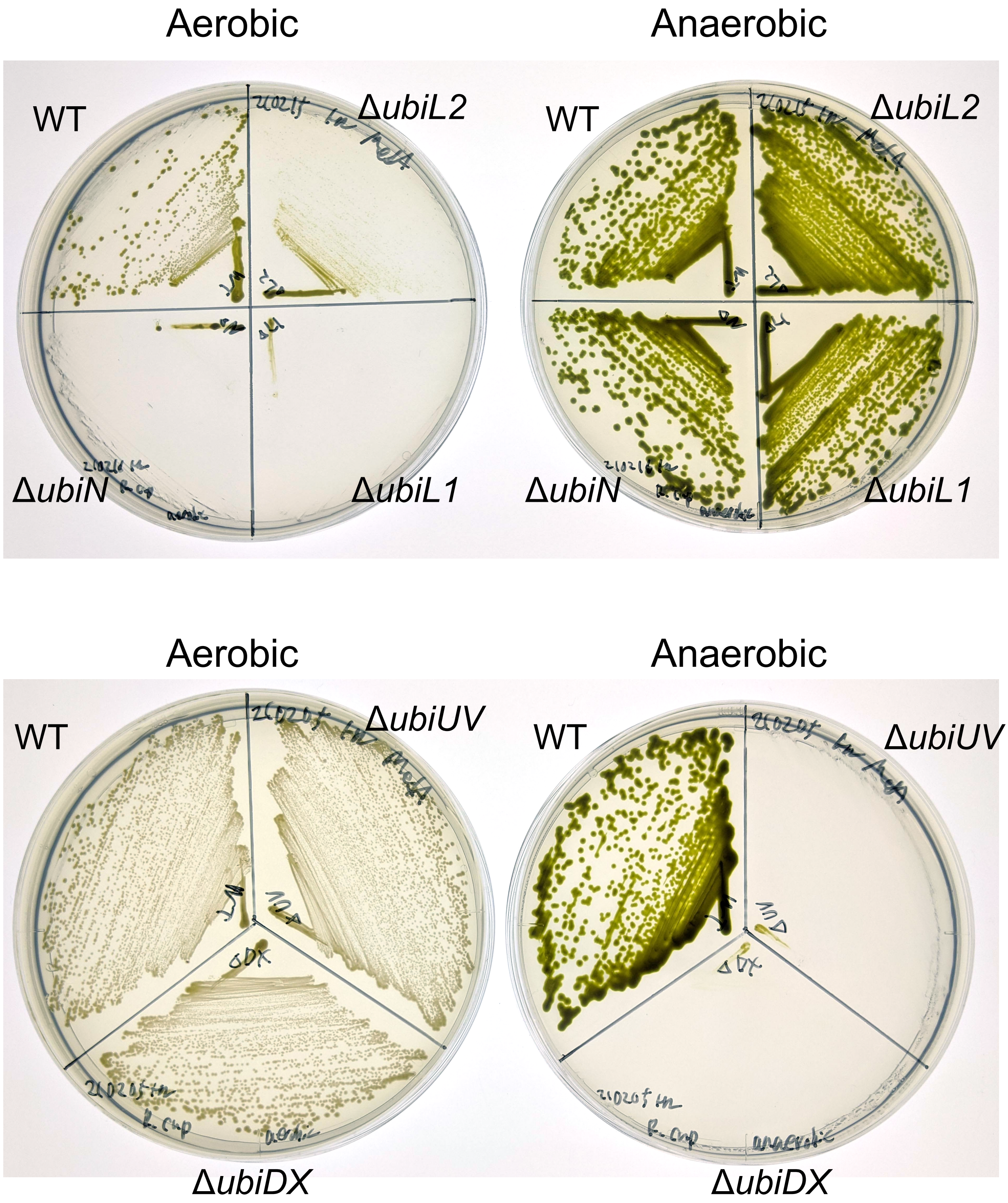

FIGURE S6. Growth test of *R. capsulatus* KO strains.

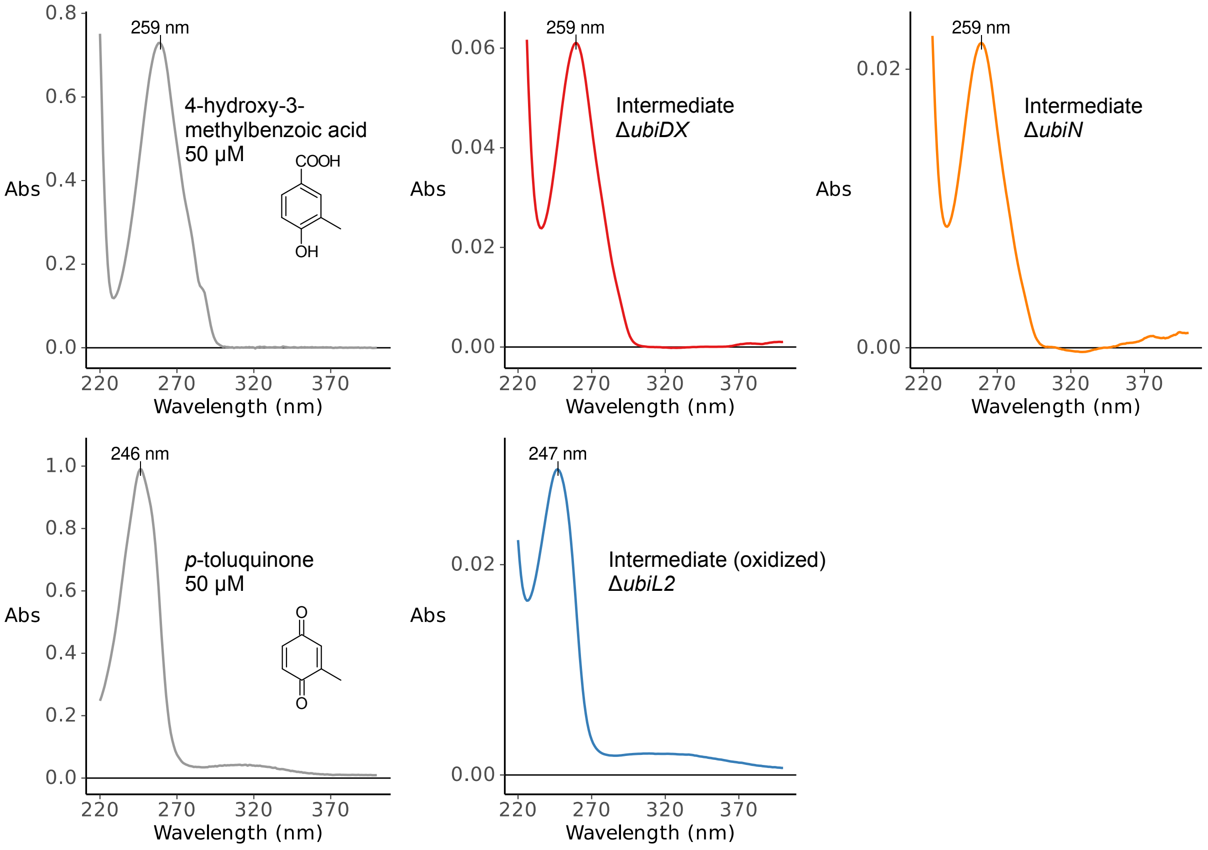

FIGURE S7. UV spectrum of UQ-biosynthetic intermediates and standards.
