# Supplementary material for: UbiN, a novel Rhodobacter capsulatus decarboxylative hydroxylase involved in aerobic ubiquinone biosynthesis
Source: FEBS Open Bio. 2023 Sep 27;13(11):2081–93. doi: 10.1002/2211-5463.13707 (PMC10626278; doi:10.1002/2211-5463.13707)
Supplement: Supplementary file 2 — Table S1. Bacterial strains and relevant genotypes. Table S2. Primers used in this study. Table S3. Plasmids used in this study. Table S4. List of FMOs used for the phylogenetic analysis. [file FEB4-13-2081-s001.docx]

**SUPPORTING INFORMATION**

**TABLE S1. Bacterial strains and relevant genotypes**

| **Strain** | **Relevant genotype and description** |
| --- | --- |
| ***E. coli*** |  |
| HB101 | *F^−^ hsdS20(rB^−^ ,mB^−^ ) recA13 supE44 proA2 leu* |
| S17-1(λpir) | *recA hsdR-M+RP4: 2-Tc:Mu: Km Tn7 λpir* |
| BW25113 | *rrnB DElacZ4787 HsdR514 DE(araBAD)567 DE(rhaBAD)568 rph-1* |
| Δ*ubiD-H* | Δ*ubiD::kan,* Δ*ubiH,* double KO strain |
| Δ*ubiH* | Δ*ubiH* |
| ***R. rubrum*** |  |
| S1 | wild type, parental strain |
| Δ*ubiL* | Δ*ubiL::kan* |
| Δ*coq7* | Δ*coq7::kan* |
| ***R. capsulatus*** |  |
| MT1131 | *crtD121*, wild type, parental strain |
| Δ*ubiL1* | Δ*ubiL1::kan* |
| Δ*ubiL2* | Δ*ubiL2::kan* |
| Δ*ubiN* | Δ*ubiN::kan* |
| Δ*ubiD-X* | Δ*ubiD-X::kan*, double KO strain |
| Δ*ubiU-V* | Δ*ubiU-V::kan*, double KO strain |

**TABLE S2. Primers used in this study**

| **Name** | **Sequence** | **Purpose** |
| --- | --- | --- |
| Fwd-XbaI-ubiL1 | CTCTCTAGAGGTGATCGTCGCCTCCTATCTG | Cloning *ubiL1* |
| Rev-ubiL1-KpnI | GAGGGTACCGGTGCGGGAAAACATGGAAA | Cloning *ubiL1* |
| Rev-ubiL1-blunt | ACCCTGTCAGCGCATCCCCAAG | Cloning *ubiL1* |
| Fwd-XbaI-ubiL2 | TAATCTAGAAGACCCAGCTTGACCGAGAC | Cloning *ubiL2* |
| Rev-ubiL2-KpnI | TTAGGTACCGACCGATTTTCCCCGCGACA | Cloning *ubiL2* |
| Rev-ubiL2-blunt | ACCCTGTCAGCGCATCCCCAAG | Cloning *ubiL2* |
| Fwd-XbaI-ubiN | GCATCTAGATTCACCGCCCATTCGAGCATC | Cloning *ubiN* |
| Rev-ubiN-KpnI | ATTGGTACCATGTCCCGGTCTGGATCGATC | Cloning *ubiN* |
| Rev-ubiN-blunt | TGTCCCGGTCTGGATCGATC | Cloning *ubiN* |
| Fwd-XbaI-ubiDX | TAATCTAGAAGTCTCCTGCCAGCGTATCGA | Cloning *ubiDX* |
| Rev-ubiDX-KpnI | ATTGGTACCTTCTGGTGATGGCGAAGATG | Cloning *ubiDX* |
| Fwd-XbaI-ubiUV | TAATCTAGAAGTTCCGGCACCTGATCGAG | Cloning *ubiUV* |
| Rev-ubiUV-KpnI | ATTGGTACCGCACCGATCGCCAGAAAGAC | Cloning *ubiUV* |
| del1-ubiL1-HindIII | ATAAAGCTTCGCGCTTTATGCGTTCAAG | Deletion *ubiL1* |
| del2-ubiL1-HindIII | TATAAGCTTATCGACGCAGATCGTGGTG | Deletion *ubiL1* |
| del1-ubiL2-HindIII | ATAAAGCTTGGCGGCAGATCTAATCGAG | Deletion *ubiL2* |
| del2-ubiL2-HindIII | TATAAGCTTGTCGTGAACCTGTGTCATGC | Deletion *ubiL2* |
| del1-ubiN-HindIII | ATAAAGCTTTCGACTGGGTCTATGGCCACGA | Deletion *ubiN* |
| del2-ubiN-HindIII | TATAAGCTTTCGGCCTGTTCCAGCACCGT | Deletion *ubiN* |
| del1-ubiDX-HindIII | ATAAAGCTTCGTGCCTGCCTTTTATCTGA | Deletion *ubiDX* |
| del2-ubiDX-HindIII | TATAAGCTTGGTGGTGCCGAAAAGATTGGT | Deletion *ubiDX* |
| del1-ubiUV-HindIII | ATAAAGCTTTCGTCTGGCAGGCAAGGTCT | Deletion *ubiUV* |
| del2-ubiUV-HindIII | TATAAGCTTGCGGTCAGTTCCTCGGTCGA | Deletion *ubiUV* |
| Fwd-RrubiL-XbaI | AATTCTAGACCCAGATAGGGCAGATAAAGC | Cloning *R. rubrum ubiL* |
| Rev-RrubiL-KpnI | TTAGGTACCGCGACCGTGGATGATTGTC | Cloning *R. rubrum ubiL* |
| Fwd-Rrcoq7-XbaI | CTCTCTAGACAGTGGCGGAGCGTTGATGG | Cloning *R. rubrum coq7* |
| Rev-Rrcoq7-KpnI | GAGGGTACCAGGGAGTGACGGTGCGTTTCA | Cloning *R. rubrum coq7* |
| del1-RrubiL-HindIII | ATAAAGCTTTGCGTGGGGTTCCCTTGTAA | Deletion *R. rubrum* *ubiL* |
| del2-RrubiL-HindIII | TATAAGCTTTTATCGGCGGACCCAGTGAC | Deletion *R. rubrum* *ubiL* |
| Fwd-EcoubiD-XbaI | ATATCTAGAATAACCGCCGATGGGACTA | Cloning *E. coli ubiD* |
| Rev-EcoubiD-KpnI | ATTGGTACCCGCCCCAGTAAATGGTGATA | Cloning *E. coli ubiD* |
| del1-EcoubiD-EcoRI | ATTGAATTCTTTCATGGCGTCCATTGTAG | Deletion *E. coli ubiD* |
| del2-EcoubiD-EcoRI | ATAGAATTCAACGGTAAAAGCGCCTGAT | Deletion *E. coli ubiD* |
| Fwd-EcoubiH-XbaI | ATATCTAGATTACGCTGGCGATATTACCC | Cloning *E. coli ubiH* |
| Rev-EcoubiH-BamHI | ATTGGATCCTCTTTCAGCGTCAGGAAGGT | Cloning *E. coli ubiH* |
| del1-EcoubiH-EcoRI | ATTGAATTCTGCTTTCTCGCAGCAACCAT | Deletion *E. coli ubiH* |
| del2-EcoubiH-EcoRI | ATAGAATTCATGCAAAGTGTTGATGTAGCC | Deletion *E. coli ubiH* |
| Fwd-RcpubiN-TA | GTGCTGATCGGTCGGAAGGT | TOP TA cloning *ubiN* |
| Rev-RcpubiN-TA | CTATTTCCGCGGCGCCGTCAC | TOP TA cloning *ubiN* |
| del1-Kpn1-sacB | CGGCAAATGGTATCTGTTCACTGACTCCCG | Construction pZJD29c |
| del2-KpnI-sacB | CGGGAGTCAGTGAACAGATACCATTGCCG | Construction pZJD29c |

**TABLE S3. Plasmids used in this study**

| **Plasmid** | **Insert and description** |
| --- | --- |
| pRK415 | Broad host range vector, Tet^R^ |
| pRK415-ubiL1 | *R. capsulatus ubiL1* |
| pRK415-ubiL2 | *R. capsulatus ubiL2* |
| pRK415-ubiN | *R. capsulatus ubiN* |
| pRK415-ubiL12 | *R. capsulatus* *ubiL1* and *ubiL2* |
| pRK415-ubiNL1 | *R. capsulatus* *ubiN* and *ubiL1* |
| pRK415-ubiNL2 | *R. capsulatus* *ubiN* and *ubiL2* |
| pZJD29a | Suicide vector, *sacB*, Gm^R^ |
| pZJD29a-coq7::kan | *R. rubrum coq7::kan* |
| pZJD29c | Suicide vector, *sacB*, Gm^R^ |
| pZJD29c-ubiL::kan | *R. rubrum ubiL::kan* |
| pZJD29c-ubiL1::kan | *R. capsulatus ubiL1::kan* |
| pZJD29c-ubiL2::kan | *R. capsulatus ubiL2::kan* |
| pZJD29c-ubiN::kan | *R. capsulatus ubiN::kan* |
| pZJD29c-ubiDX::kan | *R. capsulatus ubiDX::kan* |
| pZJD29c-ubiUV::kan | *R. capsulatus ubiUV::kan* |
| pZJD29c-EcoubiD::kan | *E. coli ubiD::kan* |
| pZJD29c-EcoΔubiH | *E. coli* Δ*ubiH* |
| pTrcHis-lacZ | *lacZ*, expression control, Amp^R^ |
| pTrcHis-ubiN | *R. capsulatus ubiN* |
| pRK2013 | Conjugation helper vector, Km^R^ |
| pHP45Ω-Km | Donor of kanamycin resistance cassette, Km^R^ |

**TABLE S4. List of FMOs used for the phylogenetic analysis**

**UbiN containing clade**

(11 proteins highlighted with orange in the tree)

WP_023911672.1 FAD-dependent monooxygenase [Rhodobacter capsulatus]

ABL68658.1 monooxygenase, FAD-binding protein [Paracoccus denitrificans PD1222]

WP_003811476.1 FAD-dependent monooxygenase [Bordetella bronchiseptica]

WP_003815139.1 MULTISPECIES: FAD-dependent monooxygenase [Bordetella]

WP_011238987.1 FAD-dependent monooxygenase [Aromatoleum aromaticum]

WP_011804579.1 FAD-dependent monooxygenase [Acidovorax sp. JS42]

WP_013519877.1 FAD-dependent monooxygenase [Alicycliphilus denitrificans]

WP_013660111.1 FAD-dependent monooxygenase [Marinomonas mediterranea]

WP_019857399.1 FAD-dependent monooxygenase [Mesorhizobium loti]

WP_021006183.1 FAD-dependent monooxygenase [Variovorax paradoxus]

WP_021007806.1 FAD-dependent monooxygenase [Variovorax paradoxus]

NP_251277.1 2-heptyl-3-hydroxy-4(1H)-quinolone synthase [Pseudomonas aeruginosa PAO1]

NP_252018.1 FAD-dependent monooxygenase [Pseudomonas aeruginosa PAO1]

NP_252879.1 monooxygenase [Pseudomonas aeruginosa PAO1]

NP_252907.1 hypothetical protein PA4217 [Pseudomonas aeruginosa PAO1]

NP_636661.1 oxidoreductase [Xanthomonas campestris pv. campestris str. ATCC 33913]

NP_636923.1 kynurenine 3-monooxygenase [Xanthomonas campestris pv. campestris str. ATCC 33913]

ABL69594.1 monooxygenase, FAD-binding protein [Paracoccus denitrificans PD1222]

ABL69603.1 monooxygenase, FAD-binding protein [Paracoccus denitrificans PD1222]

ABL71680.1 monooxygenase, FAD-binding protein [Paracoccus denitrificans PD1222]

EIL87952.1 monooxygenase FAD-binding protein [Rhodanobacter fulvus Jip2]

EIL88490.1 2-polyprenyl-6-methoxyphenol hydroxylase-like oxidoreductase [Rhodanobacter fulvus Jip2]

AFK52095.1 2-polyprenyl-6-methoxyphenol hydroxylase [Tistrella mobilis KA081020-065]

AFK54964.1 3-hydroxybenzoate 6-hydroxylase [Tistrella mobilis KA081020-065]

WP_010411973.1 NAD(P)/FAD-dependent oxidoreductase [Citromicrobium sp. JLT1363]

WP_010412122.1 FAD-dependent oxidoreductase [Citromicrobium sp. JLT1363]

WP_010926295.1 FAD-dependent monooxygenase [Bordetella bronchiseptica]

WP_010926630.1 FAD binding domain-containing protein [Bordetella bronchiseptica]

WP_011236599.1 3-hydroxybenzoate 6-monooxygenase [Aromatoleum aromaticum]

WP_011945995.1 NAD(P)/FAD-dependent oxidoreductase [Legionella pneumophila]

WP_012112099.1 FAD-dependent oxidoreductase [Parvibaculum lavamentivorans]

WP_012511559.1 NAD(P)/FAD-dependent oxidoreductase [Stenotrophomonas maltophilia]

WP_012511802.1 NAD(P)/FAD-dependent oxidoreductase [Stenotrophomonas maltophilia]

WP_013206876.1 flavin-dependent oxidoreductase [Ralstonia solanacearum]

WP_013206913.1 NAD(P)/FAD-dependent oxidoreductase [Ralstonia solanacearum]

WP_013659945.1 FAD-dependent monooxygenase [Marinomonas mediterranea]

WP_013660705.1 flavin-dependent oxidoreductase [Marinomonas mediterranea]

WP_013662918.1 FAD-dependent urate hydroxylase HpxO [Marinomonas mediterranea]

WP_019860100.1 MULTISPECIES: NAD(P)/FAD-dependent oxidoreductase [Mesorhizobium]

WP_019861478.1 flavin-dependent oxidoreductase [Mesorhizobium loti]

WP_019862452.1 NAD(P)/FAD-dependent oxidoreductase [Mesorhizobium loti]

WP_020590502.1 FAD-dependent monooxygenase [Kiloniella laminariae]

WP_021005115.1 3-hydroxybenzoate 6-monooxygenase [Variovorax paradoxus]

WP_021012742.1 NAD(P)/FAD-dependent oxidoreductase [Variovorax paradoxus]

WP_027047904.1 FAD-dependent monooxygenase [Mesorhizobium loti]

WP_040349213.1 NAD(P)/FAD-dependent oxidoreductase [Brevundimonas sp. BAL3]

WP_051255291.1 flavin-dependent oxidoreductase [Variovorax paradoxus]

WP_080643958.1 NAD(P)/FAD-dependent oxidoreductase [Mesorhizobium loti]

WP_081611334.1 NAD(P)/FAD-dependent oxidoreductase [Kordiimonas gwangyangensis]

WP_083799768.1 FAD-dependent monooxygenase [Marinomonas mediterranea]

**UbiL**WP_013066967.1 UbiH/UbiF family hydroxylase [Rhodobacter capsulatus]

WP_013069027.1 UbiH/UbiF/VisC/COQ6 family ubiquinone biosynthesis hydroxylase [Rhodobacter capsulatus]

YP_428788.1 2-octaprenyl-3-methyl-6-methoxy-1,4-benzoquinol hydroxylase [Rhodospirillum rubrum ATCC 11170]

ABL70757.1 2-octaprenyl-3-methyl-6-methoxy-1,4-benzoquinol hydroxylase / 2-octaprenyl-6-methoxyphenol hydroxylase [Paracoccus denitrificans PD1222]

ABL72008.1 Ubiquinone biosynthesis hydroxylase, UbiH/UbiF/VisC/COQ6 family [Paracoccus denitrificans PD1222]

ACJ00155.1 2-polyprenyl-6-methoxyphenol 4-hydroxylase, putative [Rhodospirillum centenum SW]

ADM08903.1 UbiH/COQ6 monooxygenase family protein [Parvularcula bermudensis HTCC2503]

EGD57818.1 2-octaprenyl-3-methyl-6-methoxy-1,4-benzoquinol hydroxylase [Novosphingobium nitrogenifigens DSM 19370]

AFK54975.1 Ubiquinone biosynthesis hydroxylase, UbiH/UbiF/VisC/COQ6 [Tistrella mobilis KA081020-065]

AFW01024.1 FAD-dependent monooxygenase [Gluconobacter oxydans H24]

WP_006013756.1 MULTISPECIES: 2-octaprenyl-6-methoxyphenyl hydroxylase [Wolbachia]

WP_007437056.1 UbiH/UbiF/VisC/COQ6 family ubiquinone biosynthesis hydroxylase [Acetobacteraceae bacterium AT-5844]

WP_008260365.1 MULTISPECIES: UbiH/UbiF/VisC/COQ6 family ubiquinone biosynthesis hydroxylase [Brevundimonas]

WP_010410420.1 FAD-dependent monooxygenase [Citromicrobium sp. JLT1363]

WP_011154692.1 FAD-dependent monooxygenase [Ehrlichia ruminantium]

WP_012110202.1 FAD-dependent monooxygenase [Parvibaculum lavamentivorans]

WP_019858090.1 UbiH/UbiF family hydroxylase [Mesorhizobium loti]

WP_019861993.1 MULTISPECIES: ubiquinone biosynthesis hydroxylase [Mesorhizobium]

WP_020398587.1 UbiH/UbiF/VisC/COQ6 family ubiquinone biosynthesis hydroxylase [Kordiimonas gwangyangensis]

WP_020590378.1 UbiH/UbiF/VisC/COQ6 family ubiquinone biosynthesis hydroxylase [Kiloniella laminariae]

CDL00515.1 putative 2-polyprenyl-6-methoxyphenol hydroxylase and related FAD-dependent oxidoreductases (UbiH) [Magnetospirillum gryphiswaldense MSR-1 v2]

WP_041404768.1 2-octaprenyl-6-methoxyphenyl hydroxylase [Rickettsia massiliae]

WP_083921868.1 FAD-dependent monooxygenase [Kiloniella laminariae]

**UbiM**

NP_637995.1 hypothetical protein XCC2647 [Xanthomonas campestris pv. campestris str. ATCC 33913]

EAR59978.1 hypothetical protein MED92_02701 [Oceanospirillum sp. MED92]

ACI98597.1 Ubiquinone biosynthesis hydroxylase, UbiH [Rhodospirillum centenum SW]

EFH10994.1 ubiquinone biosynthesis hydroxylase, UbiH/UbiF/VisC/COQ6 family [Roseomonas cervicalis ATCC 49957]

EFM05404.1 ubiquinone biosynthesis hydroxylase, UbiH/UbiF/VisC/COQ6 family [Neisseria meningitidis ATCC 13091]

EGC16894.1 ubiquinone biosynthesis hydroxylase, UbiH/UbiF/VisC/COQ6 family [Kingella denitrificans ATCC 33394]

EGD59198.1 hypothetical protein Y88_1260 [Novosphingobium nitrogenifigens DSM 19370]

EIL92972.1 hypothetical protein UU9_00605 [Rhodanobacter fulvus Jip2]

AFK54292.1 hypothetical protein TMO_2454 [Tistrella mobilis KA081020-065]

EFG30291.2 UbiH/UbiF/VisC/COQ6 family ubiquinone biosynthesis hydroxylase [Simonsiella muelleri ATCC 29453]

EKF82976.1 ubiquinone biosynthesis hydroxylase [Moraxella catarrhalis RH4]

AFW01459.1 hypothetical protein B932_1891 [Gluconobacter oxydans H24]

WP_004150679.1 5-demethoxyubiquinol-8 5-hydroxylase UbiM [Stenotrophomonas maltophilia]

WP_005674913.1 5-demethoxyubiquinol-8 5-hydroxylase UbiM [Lautropia mirabilis]

WP_007436340.1 5-demethoxyubiquinol-8 5-hydroxylase UbiM [Acetobacteraceae bacterium AT-5844]

WP_008263583.1 MULTISPECIES: 5-demethoxyubiquinol-8 5-hydroxylase UbiM [Brevundimonas]

WP_010412178.1 5-demethoxyubiquinol-8 5-hydroxylase UbiM [Citromicrobium sp. JLT1363]

WP_011803522.1 5-demethoxyubiquinol-8 5-hydroxylase UbiM [Diaphorobacter sp. LR2014-1]

WP_012031324.1 5-demethoxyubiquinol-8 5-hydroxylase UbiM [Dichelobacter nodosus]

WP_013121880.1 5-demethoxyubiquinol-8 5-hydroxylase UbiM [Thiomonas intermedia]

WP_013148603.1 5-demethoxyubiquinol-8 5-hydroxylase UbiM [Methylotenera versatilis]

WP_013517013.1 5-demethoxyubiquinol-8 5-hydroxylase UbiM [Alicycliphilus denitrificans]

WP_014111363.1 5-demethoxyubiquinol-8 5-hydroxylase UbiM [Taylorella asinigenitalis]

WP_014428639.1 5-demethoxyubiquinol-8 5-hydroxylase UbiM [Rubrivivax gelatinosus]

WP_018025500.1 5-demethoxyubiquinol-8 5-hydroxylase UbiM [Oligella urethralis]

WP_019672444.1 5-demethoxyubiquinol-8 5-hydroxylase UbiM [Psychrobacter lutiphocae]

WP_019856453.1 MULTISPECIES: 5-demethoxyubiquinol-8 5-hydroxylase UbiM [Mesorhizobium]

WP_021003848.1 5-demethoxyubiquinol-8 5-hydroxylase UbiM [Variovorax paradoxus]

ETA83905.1 hypothetical protein HMPREF1177_01105 [Eikenella corrodens CC92I]

WP_023949944.1 5-demethoxyubiquinol-8 5-hydroxylase UbiM [Pelistega indica]

WP_040389386.1 5-demethoxyubiquinol-8 5-hydroxylase UbiM [Cardiobacterium valvarum]

WP_041928505.1 5-demethoxyubiquinol-8 5-hydroxylase UbiM [Methylotenera mobilis]

**UbiF**

NP_415195.1 3-demethoxyubiquinol 3-hydroxylase [Escherichia coli str. K-12 substr. MG1655]

EAR59540.1 monooxygenase, FAD-binding [Oceanospirillum sp. MED92]

YP_857731.1 2-octaprenyl-3-methyl-6-methoxy-1,4-benzoquinol hydroxylase [Aeromonas hydrophila subsp. hydrophila ATCC 7966]

WP_005609181.1 FAD-dependent oxidoreductase [Actinobacillus pleuropneumoniae]

WP_011394734.1 FAD-dependent monooxygenase [Hahella chejuensis]

WP_012705998.1 2-octaprenyl-3-methyl-6-methoxy-1,4-benzoquinol hydroxylase [Vibrio cholerae]

WP_014948995.1 FAD-dependent monooxygenase [Alteromonas macleodii]

WP_015465742.1 FAD-dependent monooxygenase [Psychromonas sp. CNPT3]

WP_086017286.1 FAD-dependent monooxygenase [Shewanella sp. ANA-3]

**UbiH**

NP_417383.1 2-octaprenyl-6-methoxyphenol 4-hydroxylase [Escherichia coli str. K-12 substr. MG1655]

NP_253910.1 2-octaprenyl-6-methoxyphenyl hydroxylase [Pseudomonas aeruginosa PAO1]

NP_636189.1 2-octaprenyl-6-methoxyphenyl hydroxylase [Xanthomonas campestris pv. campestris str. ATCC 33913]

EAR61114.1 2-octaprenyl-6-methoxyphenyl hydroxylase [Oceanospirillum sp. MED92]

YP_856252.1 2-polyprenyl-6-methoxyphenol 4-hydroxylase [Aeromonas hydrophila subsp. hydrophila ATCC 7966]

ACK78396.1 2-octaprenyl-6-methoxyphenol hydroxylase [Acidithiobacillus ferrooxidans ATCC 23270]

EIL91924.1 2-octaprenyl-6-methoxyphenyl hydroxylase [Rhodanobacter fulvus Jip2]

EKF83221.1 2-octaprenyl-6-methoxyphenol hydroxylase [Moraxella catarrhalis RH4]

WP_000132411.1 2-octaprenyl-6-methoxyphenyl hydroxylase [Vibrio cholerae]

WP_003015223.1 FAD-dependent monooxygenase [Francisella tularensis]

WP_003814217.1 MULTISPECIES: UbiH/UbiF/VisC/COQ6 family ubiquinone biosynthesis hydroxylase [Bordetella]

WP_004084429.1 2-octaprenyl-6-methoxyphenyl hydroxylase [Xylella fastidiosa]

WP_005619006.1 2-octaprenyl-6-methoxyphenyl hydroxylase [Actinobacillus pleuropneumoniae]

WP_011236457.1 FAD-dependent monooxygenase [Aromatoleum aromaticum]

WP_011289388.1 FAD-dependent monooxygenase [Dechloromonas aromatica]

WP_011312845.1 FAD-dependent monooxygenase [Thiobacillus denitrificans]

WP_011395027.1 2-octaprenyl-6-methoxyphenyl hydroxylase [Hahella chejuensis]

WP_011634282.1 FAD-dependent monooxygenase [Nitrosomonas eutropha]

WP_011945283.1 2-octaprenyl-6-methoxyphenyl hydroxylase [Legionella pneumophila]

WP_012510105.1 2-octaprenyl-6-methoxyphenyl hydroxylase [Stenotrophomonas maltophilia]

WP_012972075.1 2-octaprenyl-6-methoxyphenyl hydroxylase [Allochromatium vinosum]

WP_013147299.1 FAD-dependent oxidoreductase [Methylotenera versatilis]

WP_013206950.1 UbiH/UbiF/VisC/COQ6 family ubiquinone biosynthesis hydroxylase [Ralstonia solanacearum]

WP_013292129.1 FAD-dependent oxidoreductase [Gallionella capsiferriformans]

WP_014430665.1 FAD-dependent monooxygenase [Rubrivivax gelatinosus]

WP_014950376.1 FAD-dependent monooxygenase [Alteromonas macleodii]

WP_015465961.1 2-octaprenyl-6-methoxyphenyl hydroxylase [Psychromonas sp. CNPT3]

WP_015831538.1 FAD-dependent monooxygenase [Methylotenera mobilis]

ERJ20799.1 UbiH protein [Salinisphaera shabanensis E1L3A]

WP_026348975.1 UbiH/UbiF/VisC/COQ6 family ubiquinone biosynthesis hydroxylase [Psychrobacter lutiphocae]

WP_041412799.1 2-octaprenyl-6-methoxyphenyl hydroxylase [Shewanella sp. ANA-3]

WP_081432175.1 2-octaprenyl-6-methoxyphenyl hydroxylase [Halorhodospira halophila]

**UbiI**

NP_417382.1 2-octaprenylphenol 6-hydroxylase [Escherichia coli str. K-12 substr. MG1655]

NP_253908.1 2-octaprenyl-3-methyl-6-methoxy-1,4-benzoquinol hydroxylase [Pseudomonas aeruginosa PAO1]

NP_636190.1 2-octaprenyl-3-methyl-6-methoxy-1,4-benzoquinol hydroxylase [Xanthomonas campestris pv. campestris str. ATCC 33913]

EAR61113.1 2-octaprenyl-3-methyl-6-methoxy-1,4-benzoquinol hydroxylase [Oceanospirillum sp. MED92]

YP_856253.1 2-octaprenyl-6-methoxyphenol hydroxylase [Aeromonas hydrophila subsp. hydrophila ATCC 7966]

ACK78157.1 2-octaprenyl-6-methoxyphenol hydroxylase [Acidithiobacillus ferrooxidans ATCC 23270]

EIL91923.1 2-octaprenyl-3-methyl-6-methoxy-1,4-benzoquinol hydroxylase [Rhodanobacter fulvus Jip2]

EKF83694.1 2-octaprenyl-3-methyl-6-methoxy-1,4-benzoquinol hydroxylase [Moraxella catarrhalis RH4]

WP_000983782.1 FAD-dependent 2-octaprenylphenol hydroxylase [Vibrio cholerae]

WP_003015224.1 UbiH/UbiF/VisC/COQ6 family ubiquinone biosynthesis hydroxylase [Francisella tularensis]

WP_003815378.1 MULTISPECIES: UbiH/UbiF family hydroxylase [Bordetella]

WP_004084431.1 UbiH/UbiF family hydroxylase [Xylella fastidiosa]

WP_005675138.1 FAD-dependent oxidoreductase [Lautropia mirabilis]

WP_011238131.1 UbiH/UbiF family hydroxylase [Aromatoleum aromaticum]

WP_011289235.1 UbiH/UbiF family hydroxylase [Dechloromonas aromatica]

WP_011312844.1 UbiH/UbiF family hydroxylase [Thiobacillus denitrificans]

WP_011634736.1 MULTISPECIES: UbiH/UbiF family hydroxylase [Nitrosomonas]

WP_011718289.1 FAD-dependent oxidoreductase [Shewanella sp. ANA-3]

WP_011806715.1 MULTISPECIES: FAD-dependent monooxygenase [unclassified Diaphorobacter]

WP_012510106.1 UbiH/UbiF family hydroxylase [Stenotrophomonas maltophilia]

WP_012972076.1 UbiH/UbiF/VisC/COQ6 family ubiquinone biosynthesis hydroxylase [Allochromatium vinosum]

WP_013124173.1 FAD-dependent monooxygenase [Thiomonas intermedia]

WP_013147300.1 FAD-dependent monooxygenase [Methylotenera versatilis]

WP_013294578.1 UbiH/UbiF family hydroxylase [Gallionella capsiferriformans]

WP_013520446.1 FAD-dependent monooxygenase [Alicycliphilus denitrificans]

WP_013659887.1 UbiH/UbiF/VisC/COQ6 family ubiquinone biosynthesis hydroxylase [Marinomonas mediterranea]

WP_014430352.1 FAD-dependent monooxygenase [Rubrivivax gelatinosus]

WP_014950375.1 MULTISPECIES: FAD-dependent 2-octaprenylphenol hydroxylase [Alteromonas]

WP_015831539.1 FAD-dependent monooxygenase [Methylotenera mobilis]

WP_018025923.1 FAD-dependent monooxygenase [Oligella urethralis]

WP_019673301.1 FAD-dependent monooxygenase [Psychrobacter lutiphocae]

WP_021012506.1 FAD-dependent monooxygenase [Variovorax paradoxus]

ERJ20798.1 2-octaprenyl-3-methyl-6-methoxy-14-benzoquinol hydroxylase protein [Salinisphaera shabanensis E1L3A]

WP_023951696.1 FAD-dependent monooxygenase [Pelistega indica]

WP_041771301.1 FAD-dependent monooxygenase [Psychromonas sp. CNPT3]

WP_043908192.1 UbiH/UbiF family hydroxylase [Ralstonia solanacearum]

WP_043947928.1 FAD-dependent monooxygenase [Actinobacillus pleuropneumoniae]

WP_081432176.1 FAD-dependent monooxygenase [Halorhodospira halophila]

**Outgroup**

ABL71129.1 monooxygenase, FAD-binding protein [Paracoccus denitrificans PD1222]

EGD57846.1 3-(3-hydroxyphenyl)propionate hydroxylase [Novosphingobium nitrogenifigens DSM 19370]

EIL92977.1 hypothetical protein UU9_00630 [Rhodanobacter fulvus Jip2]

WP_007436159.1 bifunctional 3-(3-hydroxy-phenyl)propionate/3-hydroxycinnamic acid hydroxylase [Acetobacteraceae bacterium AT-5844]

WP_007437232.1 FAD-dependent oxidoreductase [Acetobacteraceae bacterium AT-5844]

WP_007439601.1 NAD(P)/FAD-dependent oxidoreductase [Acetobacteraceae bacterium AT-5844]

WP_010925977.1 FAD-dependent oxidoreductase [Bordetella bronchiseptica]

WP_011995045.1 FAD-dependent monooxygenase [Parvibaculum lavamentivorans]

WP_012110448.1 bifunctional 3-(3-hydroxy-phenyl)propionate/3-hydroxycinnamic acid hydroxylase [Parvibaculum lavamentivorans]

WP_012511450.1 FAD-dependent monooxygenase [Stenotrophomonas maltophilia]

WP_020398153.1 FAD-dependent monooxygenase [Kordiimonas gwangyangensis]

WP_021012974.1 FAD-dependent oxidoreductase [Variovorax paradoxus]

WP_040290988.1 FAD-binding monooxygenase [Acetobacteraceae bacterium AT-5844]

WP_041946908.1 bifunctional 3-(3-hydroxy-phenyl)propionate/3-hydroxycinnamic acid hydroxylase [Variovorax paradoxus]

WP_080643741.1 FAD-dependent monooxygenase [Mesorhizobium loti]
